# Insights into protein synthesis dynamics of gilts from the same genetic background and age differing in protein deposition

**DOI:** 10.1038/s41598-026-52194-3

**Published:** 2026-05-15

**Authors:** Aline Remus, Marie-France Palin, Hélène Lapierre, Jaap van Milgen, Candido Pomar

**Affiliations:** 1 Sherbrooke Research and Development Centre, Agriculture and Agri-Food Canada, Sherbrooke, Québec J1M 0C8 Canada; 2 INRAe, UMR1348 Pegase, Saint-Gilles, France

**Keywords:** Fractional synthesis rate, Transcriptomics, Insulin, Glucose, IGF-I, Animal physiology, Physiology

## Abstract

Protein synthesis in Low and High protein deposition (PD) gilts, exploring regulatory pathways within the same genetic background and age were studied. Gilts in Low (157 g/d) and High (219 g/d) PD groups underwent jugular vein cannulation to assess insulin, IGF-I and glucose postprandial responses to the same nutrient intake. L[1-^13^C]valine administration enabled measuring protein synthesis rate and efficiency. Results showed 94% greater (*P* < 0.05) fractional synthesis rates in the longissimus dorsi and tended (*P* = 0.10) to a greater (11%) absolute synthesis rate in the liver of High PD gilts. High PD gilts tended (*P* = 0.10) to be more sensitive to insulin. Transcriptomics analyses in muscle identified 67 up-regulated and 102 down-regulated unique genes. Among the up-regulated genes, four olfactory receptors (OR4L1, OR5D13, OR6B2, OR10R2) and one ribosomal protein (RPS15A) present the highest fold-changes in High vs. Low PD gilts. Functional analyses identified six enriched gene ontology terms relate to muscle development, three to protein metabolism and four to signaling pathways. Rap1 signaling and regulation of actin cytoskeleton were over-represented KEGG pathways. High PD gilts exhibit greater protein synthesis and efficiency of protein synthesis, with transcriptomic evidence suggesting changes in the glucose/insulin metabolism and reduced muscle protein degradation.

**Supplementary Information:**

The online version contains supplementary material available at 10.1038/s41598-026-52194-3.

## Introduction

Protein deposition (PD), or protein accretion, is a critical process in animal physiology, representing protein accumulation within their bodies. It is intricately linked to muscle growth and development, contributing significantly to lean meat production, but also to the synthesis of essential proteins in various organs and tissues. Protein accretion rate is influenced by the genetic background, nutrition and management practices, showcasing the complex interplay of factors determining an animal protein synthesis and degradation^1^. Although studies on PD have traditionally focused primarily on body weight (BW), more recent research^1^ highlights the importance of considering multiple physiological and environmental influences, including sanitary status, feeding strategies and dietary protein supply, and thermal stress. Factors such as nutrient availability, hormonal regulation, and physical activity can stimulate protein synthesis, whereas fasting, aging, and stress tend to suppress it by modulating the signaling pathways and molecular mechanisms that regulate muscle protein synthesis^1^.

Studies^2–4^ on neonatal pigs emphasize the role of insulin in protein metabolism. In neonatal pig skeletal muscle, the stimulatory effect of insulin and amino acids (AA) on translation-initiation signaling is markedly higher in younger animals than in older ones, indicating a developmentally regulated decline in anabolic signalling capacity^4^. This effect is particularly evident in fatter pigs compared their leaner counterparts^5^, where insulin and glucose concentrations are closely associated with the regulation of muscle protein synthesis. Moreover, studies in juvenile and adult pigs show that early growth and birthweight influence later insulin sensitivity, indicating complexity beyond a simple age-decline^6^. Alongside hormonal factors, variations in mechanistic target of rapamycin complex 1 (mTORC1) activation for protein synthesis may lead to diverse responses to stimuli. Whereas some pigs may exhibit resistance to mTORC1’s growth-promoting actions, as observed in aging mice^7^, a previous study^8^ on pigs with low and high residual feed provided substantial evidence of reduced protein degradation in more growth-efficient pigs, without altering insulin signalling intermediates and translation initiation signalling proteins within the mTORC1 signaling pathway. No similar study has been conducted to assess PD rate variances in pigs receiving the same diet and having the same age and BW. When studying body composition changes in pigs fed at or above nutritional requirements^9^, we observed that pigs responded differently to the same nutrient density^10,11^. A large variation within treatments in nutritional titration studies is observed, even when genetics, age, and BW are initially controlled^10,11^. Such variation in PD becomes more evident as animals age, resulting in changes in body protein and lipid masses^5,12^. Fractional protein synthesis rate tends to decrease with increased BW, whereas protein degradation shows minimal changes in pigs^13^. In addition, variation in PD rates may result not only from differences in protein synthesis but also from reduced AA catabolism, which enhances the efficiency of AA utilization for protein accretion rather than oxidation^14^.

Given the multifactorial nature of PD, integrating complementary methodologies is essential to uncover the metabolic and regulatory mechanisms responsible for individual variation under same nutritional and genetic conditions. Transcriptomic analyses^15^ offer insights beyond the mTORC1 complex, shedding light on molecular pathways influencing PD variability. In addition, the use of labeled AA^16^ to directly measure protein synthesis allows studying factors linked to variation in protein accretion. Comprehending the factors and mechanisms influencing PD variation is pivotal for optimizing feeding strategies that decrease environmental impact and enhance meat production efficiency. This study aimed to directly measure protein synthesis in gilts with low and high PD rates and to conduct transcriptomic analyses to elucidate the metabolic pathways regulating these variations. Additionally, a plasma panel of insulin, glucose, and IGF-I was analyzed to characterize the metabolic differences between low- and high-PD gilts under identical nutrient intake conditions, offering a comprehensive description of their metabolic status.

## Results

### Growth and body composition 

No differences in initial conditions (Table 1) were observed between the Low and High PD groups, except for body lipid mass, which tended (*P* = 0.07) to be greater in the Low PD group. However, at the end of the experimental growth period (day 21), when the Low and High PD gilts were characterized and selected, the High PD gilts had greater BW, body protein mass (*P* < 0.01), and the tendency for greater body lipid mass (*P* = 0.07). Although lipid deposition (g/d) did not differ between groups, High PD gilts were leaner, with a higher (*P* = 0.02) body protein percentage. Average daily feed intake differed between groups in kg/d (*P* = 0.01). However, when intake was expressed relative to body weight, both groups consumed approximately 3.8% of their body weight in feed. The difference in BW observed at selection on day 21 was also present on day 28, the^13^ C-valine infusion day.

**Table 1:** Body weight (BW) and body composition of Low (158 g/d) and High (219 g/d) protein deposition gilts at initial conditions (day 0), at selection after receiving a diet ad libitum for 21 days, and at day 28 during that^13^ C-valine flooding infusion.

Item	Protein deposition group		MSE^1^	*P*-value
	Low	High		
*Initial conditions*				
Body weight, kg	32.6	32.5	1.44	0.90
Body protein mass, kg	5.8	5.8	0.28	0.36
Body lipid mass, kg	5.3	5.1	0.16	0.07
Body lipid mass, % of BW	15.7	16.3	0.12	0.12
Body protein mass, % of BW	17.7	17.8	0.36	0.76
*At selection*				
Body weight, kg	51.6	57.9	1.43	< 0.01
Body protein mass, kg	9.1	10.5	0.22	< 0.01
Body lipid mass, kg	9.1	9.1	0.54	0.90
Average daily feed intake, kg	1.97	2.22	0.01	0.01
Protein deposition, g/d	158	219	5.3	< 0.01
Lipid deposition, g/d	181	193	19.2	0.57
Body lipid mass, % of BW	17.4	15.8	0.66	0.07
Body protein mass, % of BW	17.6	18.0	0.35	0.02
On ^13^C infusion day				
Body weight, kg	60.4	66.6	1.81	< 0.01

^1^MSE, maximum standard error. 8 observations per treatment on initial and final conditions, and 7 observations for High and 8 for Low on infusion day.

#### Protein synthesis

 In the *longissimus dorsi* muscle, the fractional synthesis rate (FSR, %/day) was higher (94% greater, *P* = 0.01) in High PD gilts compared to Low PD gilts (Table 2), whereas, there were no differences in the jejunum (*P* = 0.70) and liver (*P* = 0.31) between the two groups. High PD gilts exhibited a 73% higher (*P* = 0.04) absolute synthesis rate (ASR, g/d) in whole body muscles and a tendency to have a 11% higher (*P* = 0.10) ASR in the liver compared to Low PD gilts. No difference was noted in the ASR of the jejunum (*P* = 0.75) between the two groups. The efficiency of protein synthesis (K_RNA_, mg/g) in the *longissimus dorsi* was 68% greater (*P* = 0.01) in High PD compared to Low PD gilts, whereas no differences were observed in the jejunum (*P* = 0.40) or liver (*P* = 0.64). There was no difference in ribosomal capacity (Cs) between low and High PD gilts (*P* > 0.10).

**Table 2:** Protein synthesis measurements and hormonal response in Low (158 g/d) and High (219 g/d) protein deposition of 64 kg body weight gilts.

Item	Protein deposition group		MSE^1^	*P*-value
	Low	High		
Number of observations	8	7		
*Fractional synthesis rate, % day*				
*Longissimus dorsi*	1.7	3.3	0.96	0.01
Jejunum	65.9	64.0	3.7	0.70
Liver	36.7	38.3	2.3	0.31
*Absolute synthesis rate, g/day*				
Whole body muscles	116	201	63.7	0.04
Jejunum	127	131	8.65	0.75
liver	91.2	101.3	4.08	0.10
*Efficiency of protein synthesis, KRNA, g/g/day*				
*Longissimus dorsi*	4.4	7.4	2.5	0.01
Jejunum	16.7	15.7	0.8	0.40
Liver	11.2	11.6	0.5	0.64
*Ribosomal capacity, Cs mg RNA/ g protein*				
*Longissimus dorsi*	3.8	4.0	0.22	0.52
Jejunum	39.6	40.9	1.0	0.36
Liver	34.4	33.0	0.9	0.28
Hormonal response^2^				
HOMA-IR	4.19	2.85	0.78	0.21
QUICKI	0.59	0.53	0.03	0.10
HOMA-%B	124	101	18.6	0.42

^1^MSE = Maximal standard error. ^2^HOMA-IR = homeostasis model assessment for estimating insulin resistance, QUICKI = quantitative insulin sensitivity check index, HOMA-%B = homeostasis model assessment for estimating β-cell function.

#### Hormonal response and insulin resistance and sensitivity

**Fig. 1:**
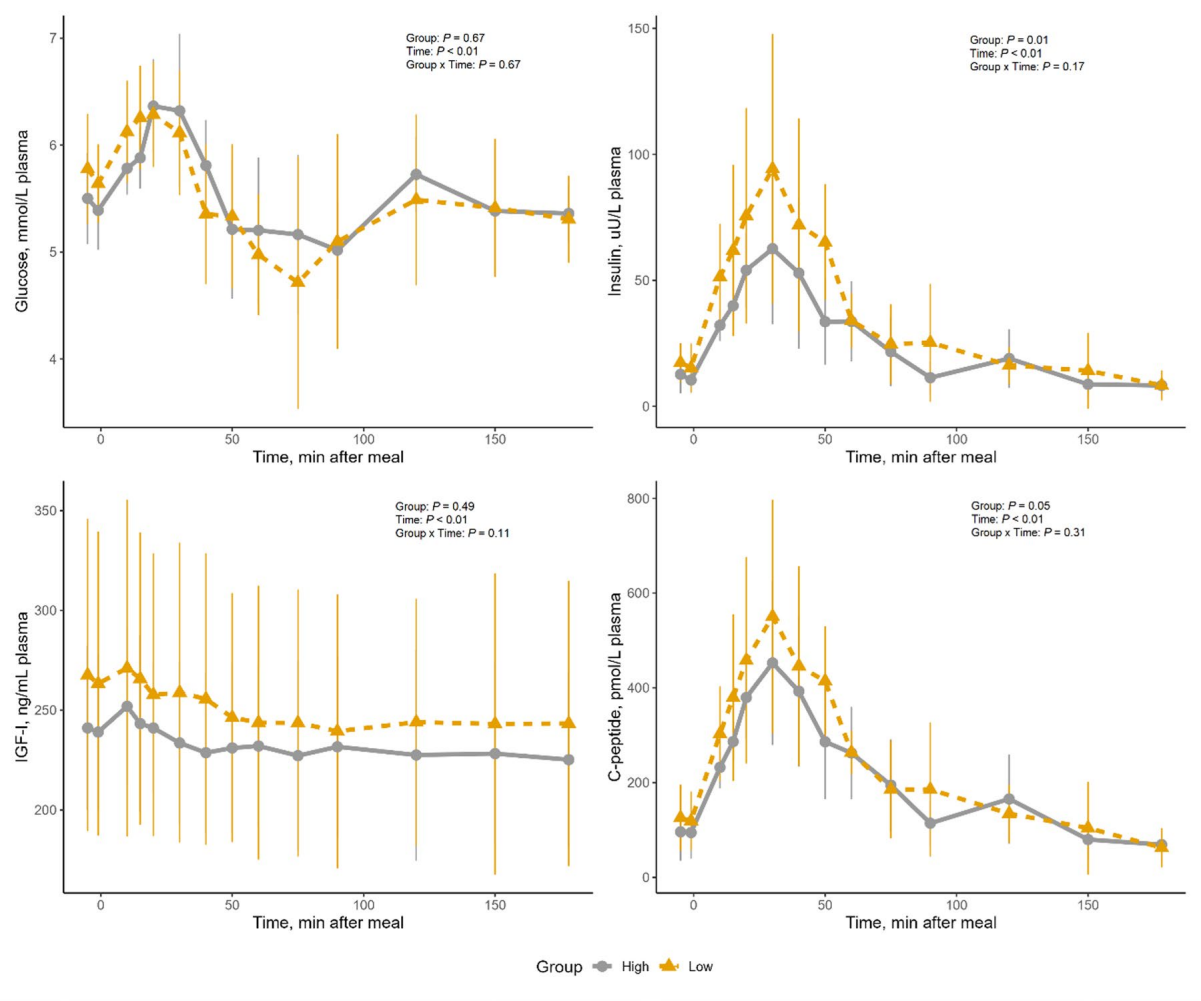
Time-course analysis of (**a**) glucose, (**b**) insulin, (**c**) IGF-1, and (**d**) C-peptide levels in relation to high (grey line) and low (orange line) protein deposition pigs at postprandial glycemic states. Vertical bars represent the standard deviation of mean values (*n* = 8).

 Plasma glucose concentration increased in response to the meal ingestion (Time: *P* < 0.01) without differences between groups (Fig. 1). Insulin and C-peptide concentrations increased after meal ingestion (Time: *P* < 0.01) and were higher (*P* ≤ 0.05) in Low than High PD pigs. IGF-1 concentrations changed over time (*P* < 0.01), with a slight post-meal increase and a gradual decrease afterwards, with no differences between PD groups. There were no differences in insulin resistance as estimated using the HOMA-IR and HOMA-%B indexes between groups (Table 2). However, insulin sensitivity estimated using QUICKI tended (*P* = 0.10) to be greater in High PD gilts compared to Low PD gilts.

#### Analysis of transcriptomic data in the longissimus muscle of High vs Low protein deposition (PD) gilts

**Fig. 2:**
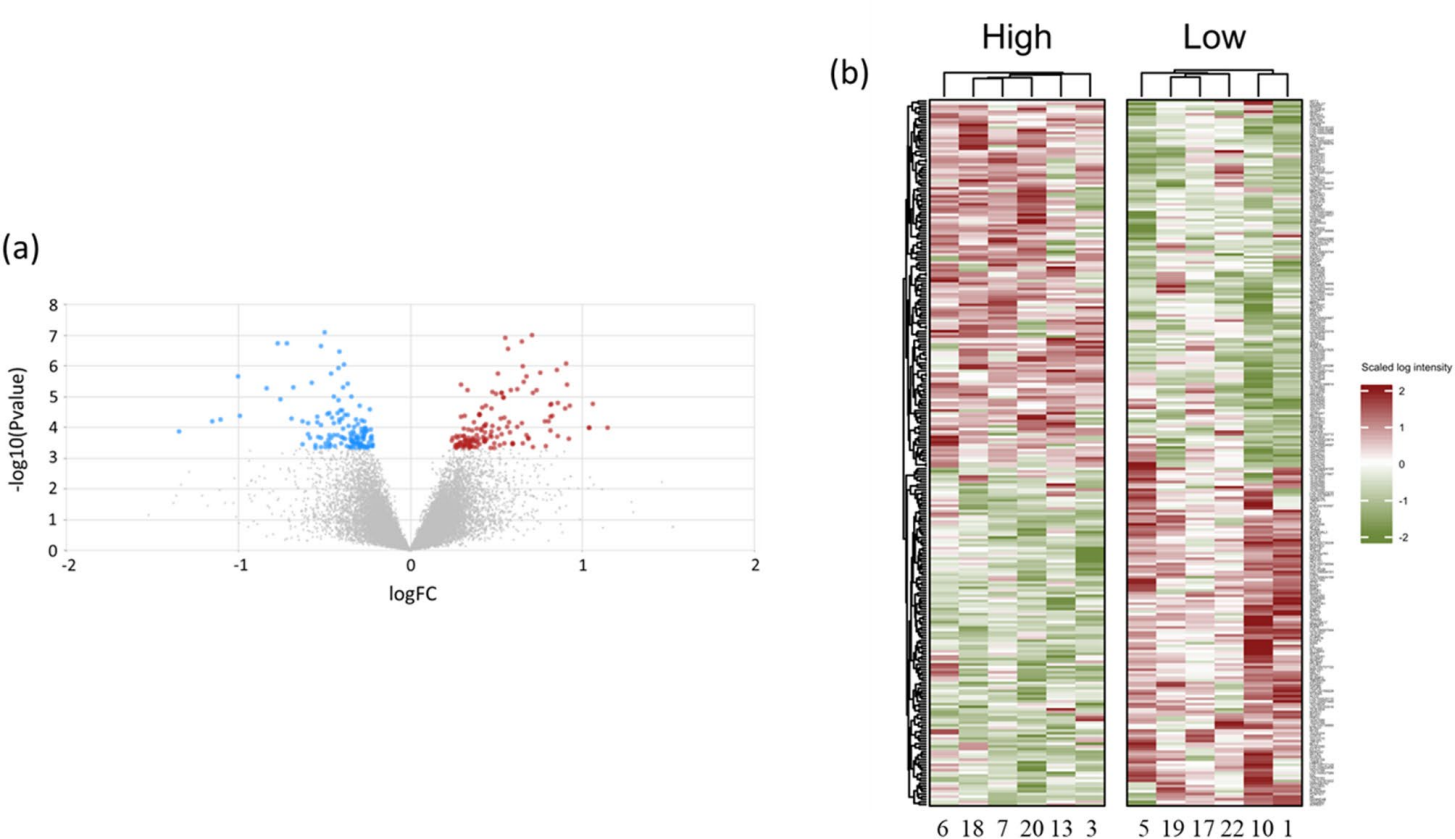
Volcano plot (**a**) and cluster heatmap (**b**) of differentially expressed genes between High and Low protein deposition (PD) gilts. (**a**) Blue and red dots represent down-regulated and up-regulated gene transcripts, respectively (FC > 1.15; FDR-adjusted *P* ≤ 0.05). Grey dots correspond to transcripts with similar expression levels between High and Low PD gilts. FC = fold change between High and Low PD gilts. (**b**) Heat map with two-way hierarchical clustering of the 277 differentially expressed gene transcripts. Each row represents one of the 277 gene transcripts and each column one of the 12 samples (*n* = 6 High and *n* = 6 Low PD gilts). Up-regulated gene transcripts are shown in red color and down-regulated in green. Color intensity reflects intensity of fold change.

A total of 277 transcripts were differentially expressed in the *longissimus* muscle of gilts with High PD relative to Low PD values (Fig. 2a, blue and red dots). Of these, 143 were up-regulated and 134 down-regulated (Fold Change (FC) > 1.15 and adjusted *P-*value ≤ 0.05, Supplementary Table S1 online). After annotation, 67 up-regulated and 102 down-regulated unique genes were identified (Supplementary Table S2 online) and, therefore, available for functional enrichment analyses. Among the list of up-regulated genes, those with the highest FC are the olfactory receptor family 4 subfamily L member 1 (*OR4L1*, FC: 1.61, *P* = 0.039), olfactory receptor family 5 subfamily D member 13 (*OR5D13*, FC: 1.66, *P* = 0.006), olfactory receptor family 6 subfamily B member 2 (*OR6B2*, FC: 1.77, *P* = 0.011), olfactory receptor family 10 subfamily R member 2 (*OR10R2*, FC: 1.76, *P* = 0.012) and the ribosomal protein S15a (*RPS15A*, FC: 1.60, *P* = 0.037). Genes that were the most down-regulated in High *vs* Low PD gilts are the pyridoxamine 5’-phosphate oxidase (*PNPO*, FC: −2.14, *P* = 0.021), serine incorporator 2 (*SERINC2*, FC: −1.64, *P* = 8.96E-04), integrin subunit alpha M (*ITGAM*, FC: −1.60, *P* = 0.006), azurocidin 1 (*AWN*, FC: −1.50, *P* = 0.037) and the stimulator of interferon response CGAMP interactor 1 (*STING1*, FC: − 1.50, *P* = 0.024).

The hierarchical clustering of all differentially expressed transcripts provided an overview of differences and similarities in the skeletal muscle transcriptional profile of gilts with High and Low PD values (Fig. 2b). The heat map dendrogram provides evidence that transcript expression profiles were subjected to clustering, with high similarity being observed within the High and Low groups and clear differences between groups.

**Fig. 3:**
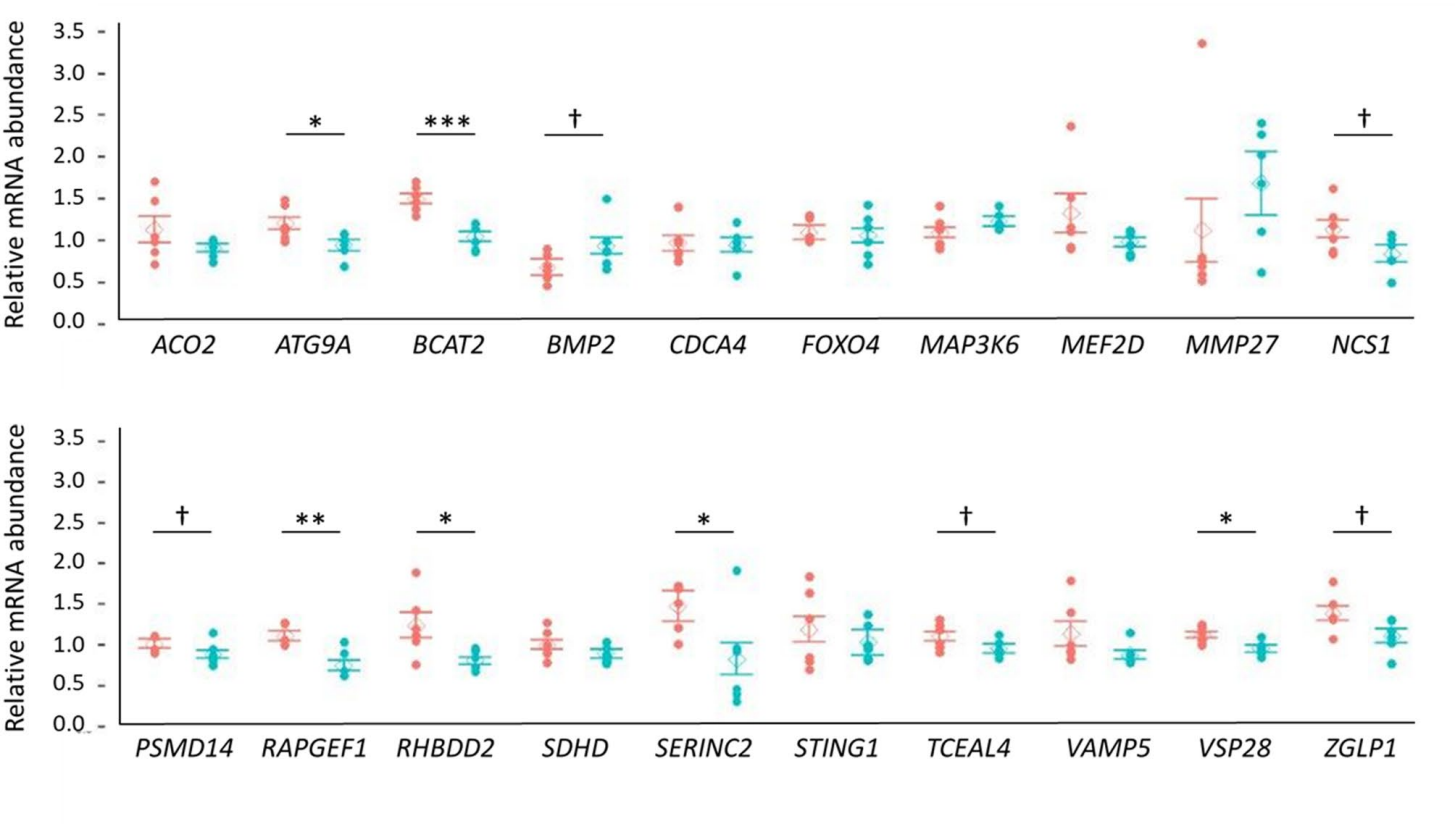
qPCR validation of selected differentially expressed genes in the *longissimus* muscle of High and Low PD gilts. Data corresponds to LS Means (lozenge center) ± 1 SEM (bars). Red dots: High PD gilts (*n* = 6); Blue dots: Low PD gilts (*n* = 6). ꝉ stands for *P* < 0.1, * for *P* ≤ 0.05, ** for *P* ≤ 0.01 and *** for *P* ≤ 0.001. Abbreviations: *ACO2*, aconitase 2; *ATG9A*, autophagy related 9 A; *BCAT2*, branched chain amino acid transaminase 2; *BMP2*, bone morphogenetic protein 2; *CDCA4*, cell division cycle associated 4; *FOXO4*, forkhead box O4; MAP3K6, mitogen-activated protein kinase kinase kinase 6; *MEF2D*, myocyte enhancer factor 2D; *MMP27*, matrix metallopeptidase 27; *NCS1*, neural calcium sensor 1; *PSMD14*, proteasome 26 S subunit, non-ATPase 14; *RAPGEF1*, rap guanine nucleotide exchange factor 1; *RHBDD2*, rhomboid domain containing 2; *SDHD*, succinate dehydrogenase complex subunit D; *SERINC2*, serine incorporator 2; *STING1*, stimulator of interferon response CGAMP interactor 1; *TCEAL4*, transcription elongation factor A like 4; *VAMP5*, vesicle associated membrane protein 5; *VPS28*, VPS28 subunit of ESCRT-I; *ZGLP1*, zinc finger GATA like protein 1.

For the qPCR validation results (Fig. 3), 13 genes (*ACO2*, *ATG9A*, *BCAT2*, *BMP2*, *MAP3K6*, *MEF2D*, *MMP27*, *RAPGEF1*, *RHBDD2*, *SERINC2*, *STING1*, *VAMP5* and *VSP28*) had the same expression trend when compared with microarray results. Of these genes, six were significantly (*ATG9A*, *BCAT2*, *RAPGEF1*, *RHBDD2*, *SERINC2* and VSP28, *P* ≤ 0.05), one tended (*BMP2*, *P* ≤ 0.10) and three were close to tendency (*MAP3K6 P* = 0.17, *MEF2D P* = 0.17, and *VAMP5 P* = 0.14) when comparing High and Low PD gilts. Four genes (*NCS1*, *PSMD14*, *TCEAL4* and *ZGLP1*) tended (*P* ≤ 0.10) to have higher mRNA abundance in Low vs. High PD gilts, which is in contrast with microarray results. The *CDCA4*, *FOXO4* and *SDHD* genes present similar mRNA abundance between the two groups of gilts.

#### Functional enrichment analyses

Biological process (BP) and KEGG pathway enrichment analyses were conducted to explore the biological significance of the 169 annotated differentially expressed genes (DEGs). A total of 20 BP gene ontology (GO) terms were significantly enriched in the High vs. Low PD gilts (Table 3, fold enrichment score (FES) > 1.5 and *P*-value < 0.05). Among these GO terms, six are related to muscle tissue development (muscle structure development (GO:0061061), muscle organ development (GO:0007517), skeletal muscle tissue development (GO:0060538), striated muscle tissue development (GO:0014706), skeletal muscle organ development (GO:0060538) and muscle tissue development (GO:0060537)) and three with protein metabolism (glycoprotein metabolic process (GO:0009100), protein modification process (GO:0036211) and regulation of protein modification process (GO:0031399)). Four GO terms include genes known to be involved in different signalling pathways (transmembrane receptor protein tyrosine kinase signalling pathway (GO:0007169), intracellular signal transduction (GO:0035556), enzyme-linked receptor protein signalling pathway (GO:0007167) and regulation of intracellular signal transduction (GO:1902531) and three are related with the response to stress (response to hypoxia (GO:0001666), response to oxygen level (GO:0070482) and cellular response to stress (GO:0033554).

**Table 3:** Over-represented Gene Ontology (GO) terms within biological process categories and enriched KEGG pathways using differentially expressed genes in the *longissimus* muscle of gilts with High vs. Low protein deposition values.

David (version 2021)	Count^1^	Gene Symbols^2^	FES^3^	*P* ^4^
GOTERM – Biological Process – ALL				
Glycoprotein metabolic process (GO:0009100)	10	*ALG12*, ***ART3***, ***BMP2***, *EXTL3*, ***FBXO2***, ***HS6ST3***, ***HYAL4***, *MAN2A2*, *SIRT2*, *TET2*	3.6	0.0019
Transmembrane receptor protein tyrosine kinase signaling pathway (GO:0007169)	10	*AKT1S1*, *AXL*, ***BMP2***, ***CHN1***, *CRKL*, *FOXO4*, *HCK*, *RAPGEF1*, *SIRT2*, *VEGFB*	2.9	0.0068
Muscle structure development (GO:0061061)	10	***BMP2***, *FOXO4*, ***GDF3***, *MEF2D*, *MYH14*, *MYLK2*, *RBFOX1*, *SIRT2*, *TMOD1*, *VAMP5*	2.9	0.0074
Muscle organ development (GO:0007517)	7	***BMP2***, *MEF2D*, *MYH14*, *MYLK2*, *RBFOX1*, *SIRT2*, *VAMP5*	3.8	0.0098
Skeletal muscle tissue development (GO:0060538)	5	*MEF2D*, *MYH14*, *MYLK2*, *RBFOX1*, *VAMP5*	5.5	0.0130
Striated muscle tissue development (GO:0014706)	7	***BMP2***, *MEF2D*, *MYH14*, *MYLK2*, *RBFOX1*, SIRT2, *VAMP5*	3.5	0.0150
Skeletal muscle organ development (GO:0060538)	5	*MEF2D*, *MYH14*, *MYLK2*, *RBFOX1*, *VAMP5*	5.2	0.0160
Anterior/posterior pattern specification (GO:0009952)	6	***BMP2***, *CRKL*, ***GDF3***, ***HOXB9***, *HOXC6*, ***HOXD13***	4.0	0.0160
Intracellular signal transduction (GO:0035556)	26	***AKAP7***, *AKT1S1*, *ARHGEF6*, *ASB7*, *ATP2A3*, *AXL*, *BCL3*, ***BMP2***, ***CHN1***, *CRKL*, ***CTF2***, *DENND4B*, *DUSP3*, *INPP5D*, *LARP1*, *MALT1*, *NDRG1*, *PPM1D*, *RAPGEF1*, *RC3H1*, ***RELL2***, ***RNF183***, ***SEZ6L2***, *SH3RF2*, *SIRT2*, T*RIM8*	1.6	0.0170
Protein modification process (GO:0036211)	34	*AKT1S1*, *ALG12*, ***ART3***, *ASB7*, ***BMP2***, *COPS7A*, *CRKL*, **CTF2**, *DUSP3*, *EIF2AK1*, *EXTL3*, ***FBXO2***, *GATAD2B*, ***GDF3***, ***GNL3***, *HCK*, *MALT1*, *MAN2A2*, *MYLK2*, *PPM1D*, ***PRDM12***, ***PSMD14***, *RAPGEF1*, *RC3H1*, ***RELL2***, *RNF10*, ***RNF183***, *SH3RF2*, *SIRT2*, *TET2*, ***TTLL8***, *VEGFB*, *VPS28*, *WBP1L*	1.5	0.0180
Cell-substrate adhesion (GO:0031589)	7	*AXL*, *CRKL*, *DUSP3*, *EPDR1*, *NTNG1*, ***RELL2***, *SORBS1*	3.3	0.0190
Muscle tissue development (GO:0060537)	7	***BMP2***, *MEF2D*, *MYH14*, *MYLK2*, *RBFOX1*, *SIRT2*, *VAMP5*	3.2	0.0220
Response to hypoxia (GO:0001666)	5	***BMP2***, *NDRG1*, ***SDHD***, SIRT2, *VEGFB*	4.6	0.0230
Enzyme linked receptor protein signaling pathway (GO:0007167)	12	*AKT1S1*, *AXL*, ***BMP2***, *BTBD11*, ***CHN1***, *CRKL*, *FOXO4*, ***GDF3***, *HCK*, *RAPGEF1*, *SIRT2*, *VEGFB*	2.1	0.0230
Response to oxygen level (GO:0070482)	5	***BMP2***, *NDRG1*, ***SDHD***, *SIRT2*, *VEGFB*	4.2	0.0300
Cell proliferation (GO:0042127)	18	***BMP2***, *CRKL*, ***CTF2***, *EIF2AK1*, *FOXO4*, *HCK*, *INPP5D*, *LARP1*, *LMBR1L*, *MALT1*, *MEF2D*, *NDRG1*, ***OTP***, *RAPGEF1*, *RC3H1*, ***RPS15A***, *SIRT2*, *VEGFB*	1.7	0.0320
Regulation of intracellular signal transduction (GO:1902531)	18	***AKAP7***, *AKT1S1*, *AXL*, *BCL3*, ***BMP2***, ***CHN1***, *CRKL*, ***CTF2***, *DENND4B*, *DUSP3*, *MALT1*, *RAPGEF1*, *RC3H1*, ***RELL2***, ***RNF183***, ***SEZ6L2***, *SH3RF2*, *TRIM8*	1.7	0.0330
Response to extracellular stimulus (GO:0009991)	6	*AXL*, *EIF2AK1*, ***GDF3***, *LARP1*, *SIRT2*, *SLC38A3*	3.1	0.0430
Cellular response to stress (GO:0033554)	19	*ATP2A3*, *BCL3*, ***BMP2***, *DUSP3*, *EIF2AK1*, ***FBXO2***, *FOXO4*, *NDRG1*, *PPM1D*, ***PSMD14***, ***RAD9B***, ***RELL2***, *RHBDD2*, ***RNF183***, *SH3RF2*, ***SDHD***, *SIRT2*, *SLC38A3*, *ZBTB40*	1.6	0.0440
Regulation of protein modification process (GO:0031399)	18	*AKT1S1*, ***BMP2***, *CRKL*, ***CTF2***, *DUSP3*, *EIF2AK1*, ***FBXO2***, ***GDF3***, ***GNL3***, *MALT1*, ***PRDM12***, *RAPGEF1*, ***RELL2***, *SH3RF2*, *SIRT2*, *VEGFB*, *VPS28*, *WBP1L*,	1.6	0.0460
KEGG pathways				
Regulation of actin cytoskeleton (04810)	6	*ARHGEF6*, *CRKL*, *ITGAM*, *MYH14*, *MYLK2*, *PFN1*	3.2	0.0370
Rap1 signaling pathway (04015)	5	*CRKL*, *ITGAM*, *PFN1*, *RAPGEF1*, *VEGFB*	2.8	0.0950

^1^Count = number of genes. ^2^Bold = up regulated genes and Plain text = down regulated genes in High vs. Low protein deposition gilts. Underlined = genes that were validated with qPCR analyses. ^3^FES = Fold Enrichment Score. ^4^Gene Ontology term enrichment *P*-value.

**Fig. 4:**
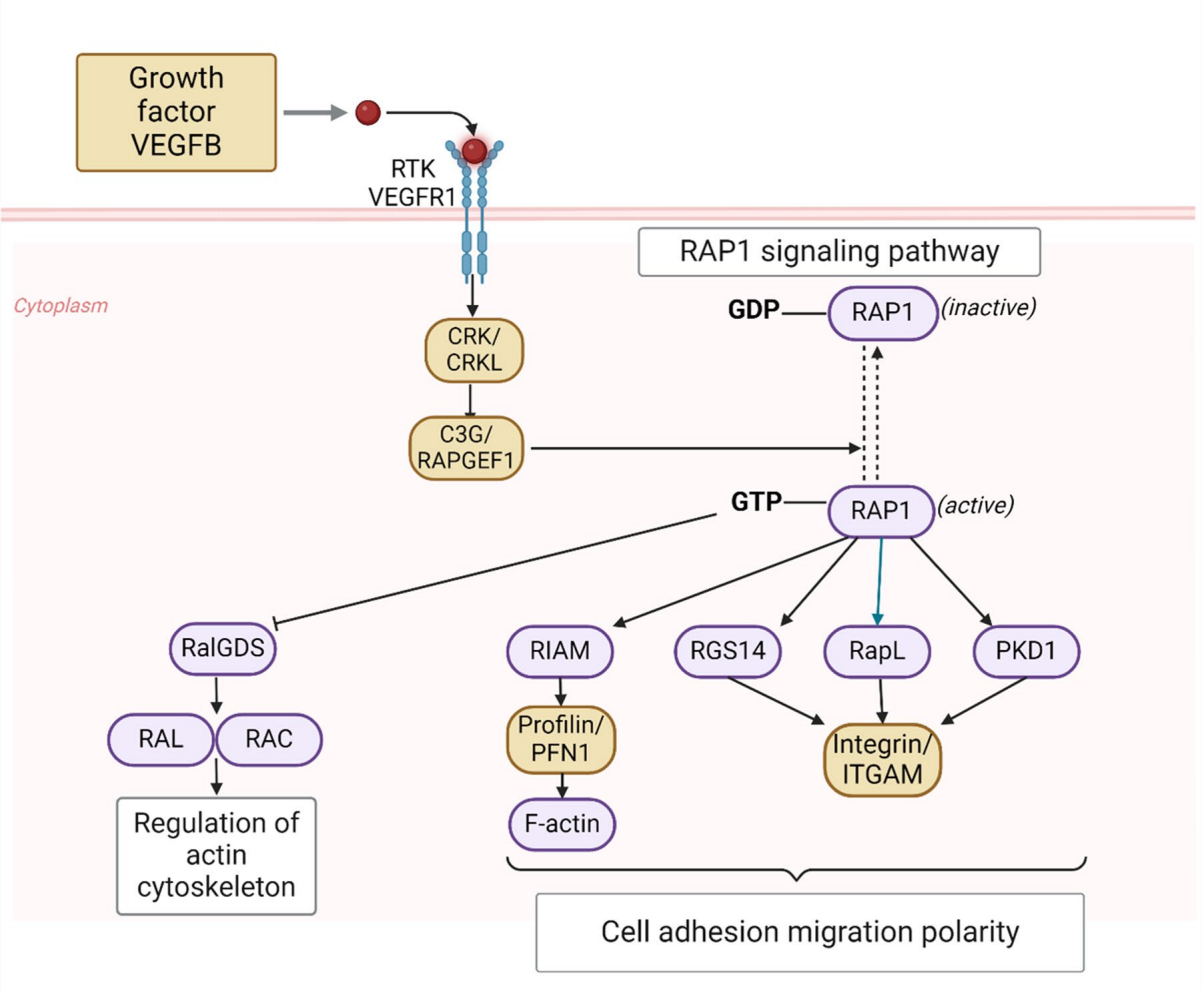
RAP1 signalling pathway from the KEGG database. This figure presents a simplified version of the RAP1 signalling pathway to better highlight interactions among differentially expressed genes (yellow boxes). This pathway is enriched when using a fold enrichment score (FES) > 1.5 and a *P*-value < 0.10. Identified DEGs (yellow) within this pathway are all down-regulated genes in High vs. Low PD gilts. *CRKL*, CRK-like proto-oncogene, adaptor protein; *ITGAM*, integrin subunit alpha M; *PFN1*, profilin 1; *RAPGEF1*, Rap guanine nucleotide exchange factor 1; *VEGFB*, vascular endothelial growth factor B. For the corresponding reference KEGG pathway: https://www.kegg.jp/pathway/hsa04015. Created in BioRender. Remus, A. (2025) https://BioRender.com/5xnhvos.

**Fig. 5:**
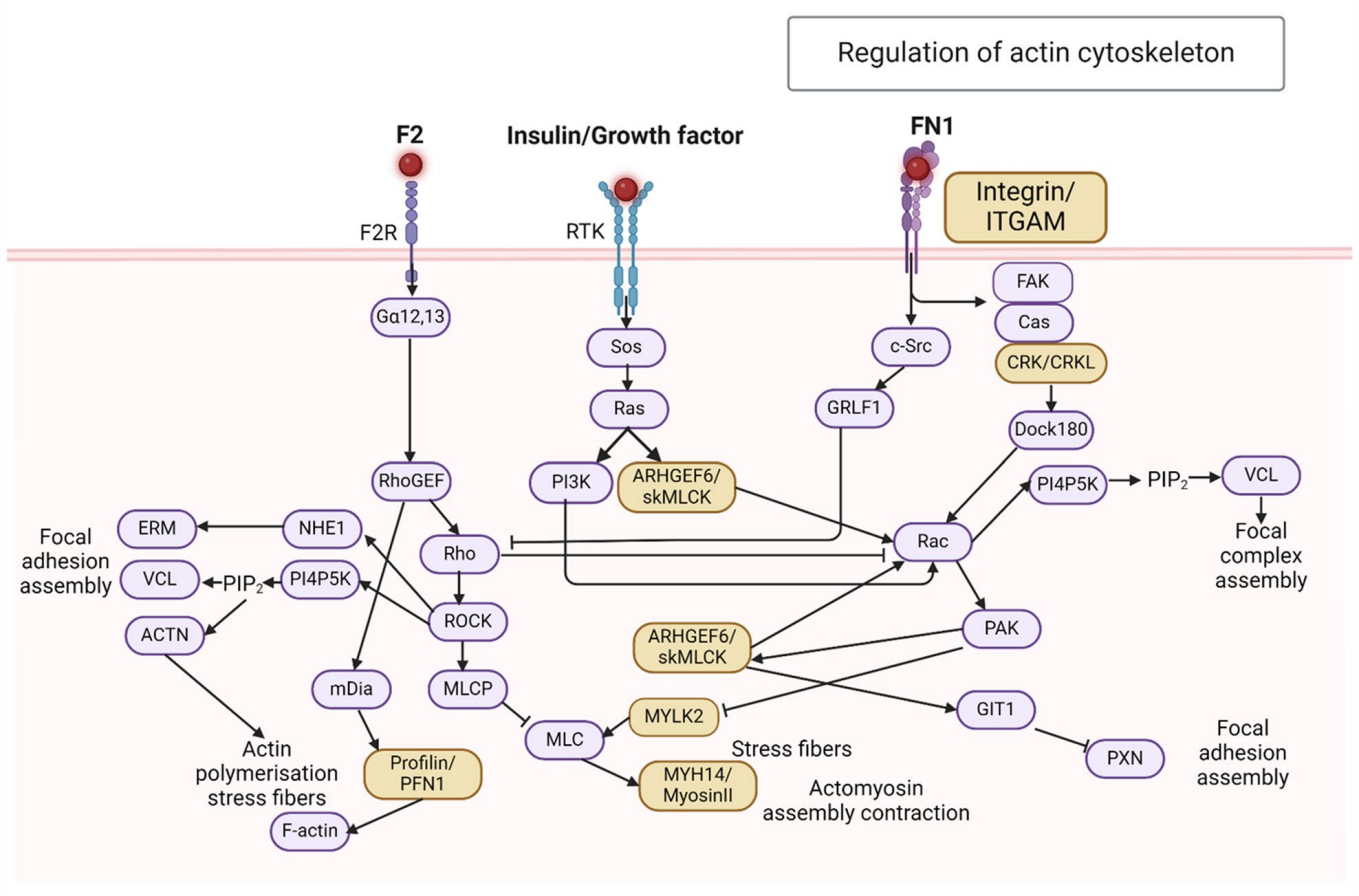
Regulation of actin cytoskeleton pathway from the KEGG database. This figure presents a simplified version of the pathway to better highlight interactions among differentially expressed genes (yellow boxes). This pathway is enriched when using a fold enrichment score (FES) > 1.5 and a *P*-value < 0.05. Identified DEGs (yellow) within this pathway are all down-regulated genes in High vs. Low PD gilts. *ARHGEF6*, Rac/Cdc42 guanine nucleotide exchange factor 6; *CRKL*, CRK like proto-oncogene, adaptor protein; *ITGAM*, integrin subunit alpha M; *MYLK2*, myosin light chain kinase 2; *MYH14*, myosin heavy chain 14; *PFN1*, profilin 1. For the corresponding reference KEGG pathway: https://www.kegg.jp/pathway/map04810. Created in BioRender. Remus, A. (2025) https://BioRender.com/0f3rg9x.

Two over-represented and interconnected KEGG pathways were identified when using a FES > 1.5 and *P*-value < 0.10 (Table 3; Rap1 signalling pathway and regulation of actin cytoskeleton). A simplified version of the Rap1 signalling and regulation of actin cytoskeleton KEGG pathways is presented in Figs. 4 and 5 to better highlight interactions among DEGs (in yellow) identified within these pathways. Please refer to https://www.kegg.jp/pathway/hsa04015 and https://www.kegg.jp/pathway/map04810 for the original Rap1 signalling and regulation of actin cytoskeleton reference pathways.

#### Protein-protein interactions (PPI) network 

**Fig. 6:**
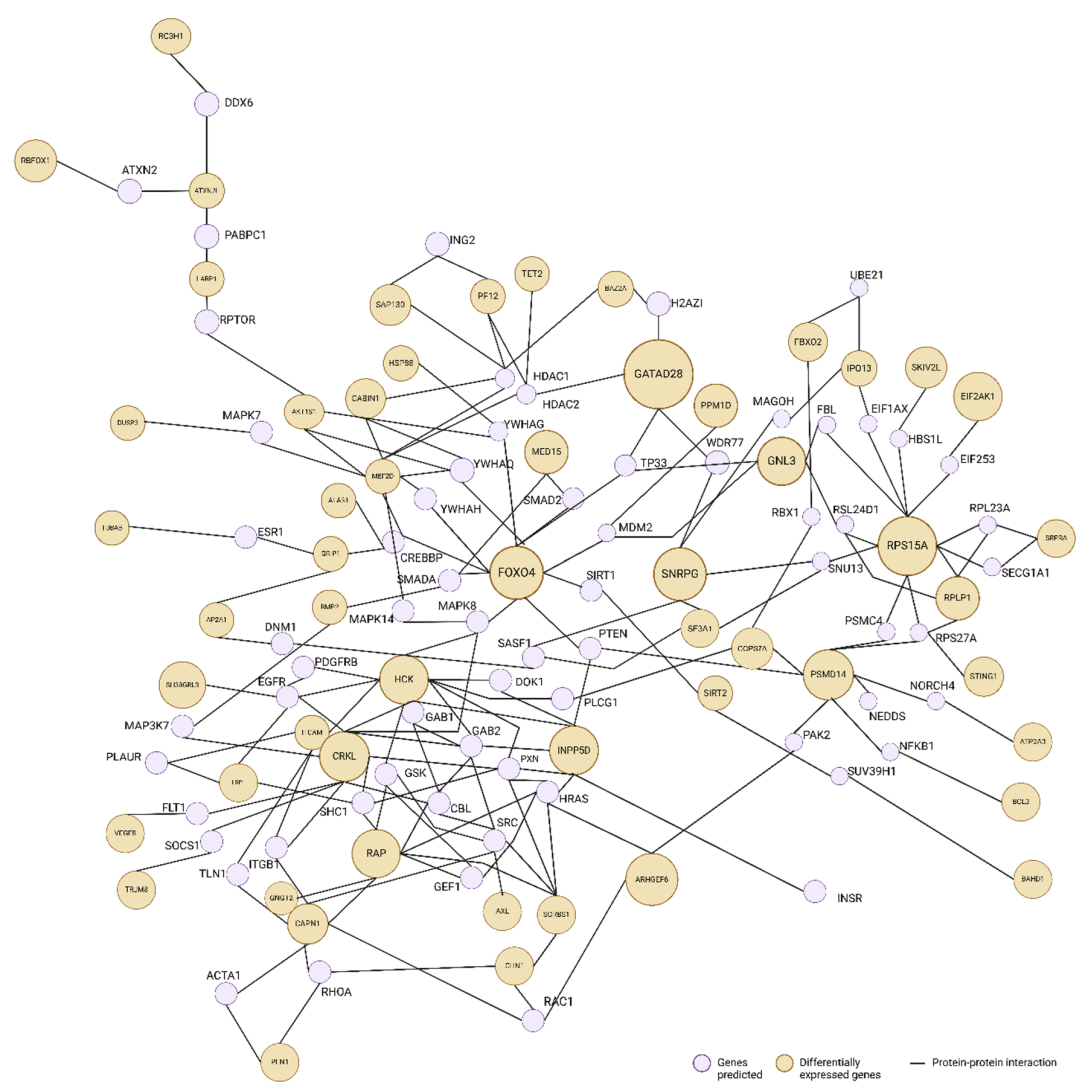
Protein-protein interaction (PPI) network was obtained from the NetworkAnalyst web-based visual analytics platform. This network includes uploaded differentially expressed genes (seed genes in yellow) in High vs. Low protein deposition gilts and genes predicted to be involved in the PPI regulatory network (non-seed genes in purple). The original image is in supplementary material figure S2. Created in BioRender. Remus, A. (2025) https://BioRender.com/jxr3hcd.

The PPI network generated by the STRING interactome database contains 121 nodes (genes/proteins, purple and yellow bubbles), including 55 seed genes (yellow bubbles) and 186 edges (Fig. 6). The node degree and betweenness centrality estimates for genes/proteins having more than 5 connections with other proteins are presented in Table 4 and include 7 seed (CRKL, HCK, RPS15A, RAPGEF1, INPP5D, MEF2D and CAPN1) and two non-seed (SRC and HDAC1) genes/proteins. The most interconnected proteins (degree values > 5) are considered as hub genes with important regulatory functions in skeletal muscle protein deposition.

**Table 4:** Centrality estimates of the protein-protein interaction (PPI) network of differentially expressed genes in the *longissimus* muscle of gilts with High vs. Low protein deposition. Hub genes showing more than 5 interactions are presented.

Node ID	Node name^1^	Degree^2^	Betweenness^3^	Proteins showing protein-protein interactions with node proteins^4^
1399	CRKL	15	1356.52	CBL, CSK, EGFR, FLT1, GAB1, GRB2, ITGB1, MAP3K7, MAPK8, MET, PXN, *RAPGEF1*, SHC1, SOCS1, SRC
3055	HCK	15	1904.98	CBL, CSK, DNM1, DOK1, EGFR, GAB1, GRB2, HRAS, *ITGAM*, MAPK8, PDGFRB, PLCG1, PXN, SRC, SRSF1
6210	**RPS15A**	11	1140.07	EIF1AX, EIF2S3, FBL, HBS1L, PSMC4, **RPLP1**, RPL23A, RPS27A, RSL24D1, SEC61A1, SNU13
2889	RAPGEF1	9	246.17	CBL, GRB2, *CRKL*, HRAS, MET, RHOA, SHC1, *SORBS1*, SRC
3635	INPP5D	8	606.43	DOK1, GAB1, GRB2, HRAS, INSR, MET, PTEN, SHC1
4209	MEF2D	7	1042.23	*AKT1S1*, BREBBP, *CABIN1*, HDAC1, HDAC2, MAPK14, YWHAH, YWHAQ
6714	SRC^5^	7	453.81	*AXL*, *ARHGEF6*, *CAPN1*, *CRKL*, **GNGT2**, *HCK*, *RAPGEF1*
823	CAPN1	7	251.57	ACTA1, CSK, ITGB1, RAC1, RHOA, SRC, TLN1
3065	HDAC1^5^	6	420.41	*BAZ2A*, *CABIN1*, *GATAD2B*, *MEF2D*, *PHF12*, *SAP130*

^1^Bold = up regulated genes and Plain text = down regulated genes in High vs. Low protein deposition gilts. CRKL, CRK like proto-oncogene, adaptor protein; HCK, HCK proto-oncogene, src family tyrosine kinase; RPS15A, ribosomal protein S15a; RAPGEF1, rap guanine nucleotide exchange factor 1; INPP5D, inositol polyphosphate-5-phosphatase D; MEF2D, myocyte enhancer factor 2D; SRC, src proto-oncogene, non-receptor tyrosine kinase; CAPN1, calpain 1; HDAC1, histone deacetylase 1; ^2^Total number of edges connected to the node. ^3^Number of shortest paths going through a node. ^4^Underlined genes/proteins corresponds to differentially expressed genes (or seed genes) in High vs. Low protein deposition gilts. ^5^Non-seed genes: genes predicted to be involved in the PPI network (NetworkAnalyst 3.0) but that were not present in the uploaded list of differentially expressed genes for High vs. Low protein deposition gilts.

#### Western blot analyses

**Fig. 7:**
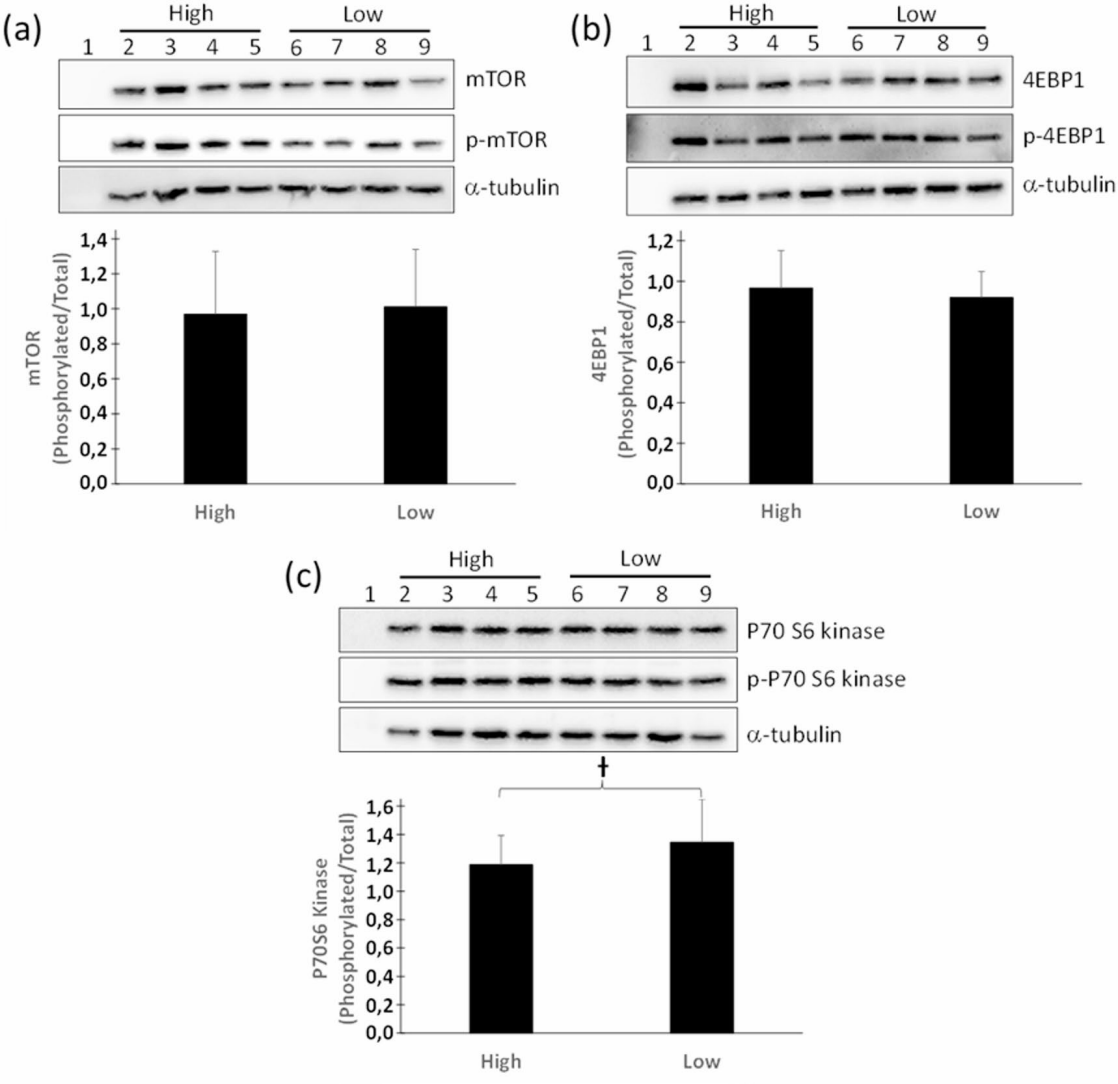
Abundance of total and phosphorylated mTOR (**a**) and its downstream effectors 4EBP1 (**b**) and P70 S6 kinase (**c**) in the longissimus muscle of gilts with High and Low protein deposition values. Phosphorylated and non-phosphorylated proteins were normalized to α-tubulin, and the resulting values were used to calculate phosphorylated/total protein ratios. A protein standard was loaded in well number 1 to confirm molecular weights (289 kDa for mTOR, 15 kDa for 4EBP1, 70 kDa for P70 S6 kinase, and 50 kDa for α-tubulin). Western blot bands shown for each target protein may originate from different parts of the same membrane and were grouped for visual clarity. Cropped sections are delineated by the black outlines. Full-length, uncropped blots corresponding to these images are provided in Supplementary Figure S1. Values are means ± SEM of *n* = 4 gilts. ꝉ indicates *P* < 0.1.

 Since mTORC1 is a critical regulator of protein synthesis and myofiber growth, western blot analyses were conducted to determine if there are differences in mTOR phosphorylation (as a proxy for mTORC1 activation) in the skeletal muscle of gilts with High or Low PD values. The activation of two mTORC1 downstream substrates known to regulate protein translation initiation and progression (P70S6K and 4EBP1) was also investigated. There was no difference in mTOR and 4EBP1 phosphorylation between the High and Low PD gilts (Fig. 7a and b), whereas gilts with Low PD values showed enhanced P70S6K phosphorylation when compared with High PD gilts (Fig. 7c; tendency, *P* = 0.07).

## Discussion

It is well known that genetic line and growth potential have a direct effect on FSR. However, the drivers for individual variation in growing animals with the same starting BW, same genetic line, and same sex are poorly characterized. We set to explore the mechanisms linked to individual variability in PD, as it is the main driver of nutrient requirements in growing animals.

In this study, High PD gilts presented a 94% greater muscle FSR, reflecting in a 58% greater ASR, and a 60% _k_RNA than Low PD gilts. As FSR is affected by the genetic line, among other factors, comparing FSR values between studies is fickle. However, using the same technique and similar BW, the average values of the present study are in line with the reference paper^16^. It seems a part of the differences in PD among pigs is due to protein synthesis efficiency, supporting the idea that some pigs may utilize nutrients more efficiently for growth than others^11^. The genetic background of an animal and the epigenetic modifications occurring during fetal development and early life can impact gene expression and, subsequently, protein metabolism. However, the molecular mechanisms involved in protein synthesis and degradation variability are poorly understood and need further characterization^8^.

It is well known that protein synthesis is supported by nutrient intake, however, differences in PD between groups could not be explained by intake alone, suggesting differences in nutrient utilization and metabolic efficiency. High PD gilts consumed more feed in absolute terms; however, when expressed relative to average BW, intake differed by only ~ 3% between groups. In contrast, PD was ~ 40% greater and nitrogen retention ~ 37% greater in High PD gilts. Importantly, the proportional increase in nitrogen retained (g/d) exceeded the increase in nitrogen intake (g/d; ~13%), and this increase in nitrogen utilization efficiency was accompanied by higher lysine, and valine efficiency and improved feed efficiency^17^. Collectively, these findings indicate that gilts with higher PD achieved greater protein accretion per unit of nutrient consumed, suggesting differences in nutrient utilization and partitioning efficiency rather than intake alone. This interpretation is consistent with previous^17,18^ work showing that gilts differing in PD did not differ in the mRNA abundance of genes involved in nutrient transport in the small intestine, indicating that post-absorptive metabolic processes likely contribute to variation in PD. Therefore, differences in PD among gilts receiving the same diet likely arise from variation in metabolic efficiency and nutrient partitioning rather than differences in nutrient supply per se. An increased secretion of anabolic hormones like insulin and IGF-1 can stimulate protein synthesis in muscle tissue^19^. Hormonal fluctuations or adaptations, even without changes in exercise or nutrition, can lead to changes in FSR. Indeed, increased insulin blood concentration was related with increased FSR^20^. The intricate interplay of metabolic and hormonal factors may elucidate the observed correlation between beta-cell function and muscle FSR in our study. Insulin, secreted by the beta-cells in response primarily to elevated blood glucose, is crucial in stimulating protein synthesis by facilitating AA uptake by various tissues, including muscle^21^. Consequently, impaired pancreatic beta-cell function and reduced insulin sensitivity may indirectly impact muscle protein synthesis. Additionally, hormones like IGF-I could further contribute to changes in FSR. In the present study, changes of IGF-I were observed only over time. Protein and caloric restriction are two known factors to affect IGF-I plasma concentrations^19^. Thus the small changes observed in IGF-I concentrations after re-feeding might be linked to the fact that pigs were previously restricted to 90% of their ad-libitum intake and fasted 10–12 h before re-feeding. This slight change might therefore be due to IGF-I returning to its basal concentration after fasting. When comparing different genetic lines, IGF-I concentration was lower in pigs having the highest protein accretion rate, thus suggesting that factors other than IGF-I would better explain differences in protein synthesis among genotypes^22^. Also, the higher bioavailability of IGF-I largely stems from dietary influences on IGF-binding proteins (IGFBPs). Elevated body mass index and insulin levels correlate with lower IGFBP-1 and IGFBP-2 concentrations, potentially elevating free IGF-I concentrations^19,23^. Although we did not observe differences in IGF-I and glucose levels between the PD groups, circulating insulin and C-peptide concentrations were notably higher in the Low PD group. Fatter-finishing pigs (22% vs. 17% body lipids content) exhibited compensatory insulin secretion in response to reduced insulin sensitivity^5^. Thus, the differences in circulating insulin and C-peptide concentrations might suggest early signs of reduced insulin sensitivity in Low PD pigs, which tended to be fatter and tended to have a lower QUICKI index than their counterparts. In contrast, lower circulating insulin in High PD gilts could reflect greater nutrient demand associated with faster lean growth rather than intrinsic differences in insulin sensitivity. It is important to note that Low and High groups were defined post-hoc based on observed PD. Thus, all gilts began the study with same body composition and body weight and were exposed to identical dietary conditions throughout the experiment. Thus, when given same conditions responded differently to nutrient supply. Although High PD gilts consumed slightly more feed in absolute terms, they exhibited substantially greater nutrient-use efficiency, including higher nitrogen efficiency and improved lysine and valine utilization (data not-shown) for protein deposition. Additionally, when expressing feed intake relative to body weight feed intake was similar (Low PD = 46.9 g/kg BW/d, High PD = 48.4 g/kg BW/d), but High PD gilts achieved greater protein deposition per unit of nutrient consumed, consistent with previous findings with the increased K_RNA_ found in this study. Although increased cellular demand associated with higher FSR likely contributes to greater nutrient utilization in High PD gilts, the disproportionate increase in nitrogen retention relative to nitrogen intake indicates that the observed differences cannot be attributed solely to demand, but also reflect improved nutrient utilization and partitioning efficiency.

No difference in jejunum and liver FSR were observed. Insulin exerts variable effects on hepatic FSR, influencing different proteins in distinct manners. However, it does not exert a direct stimulatory or inhibitory effect on the global hepatic protein synthesis processes, except in the case of albumin, where it demonstrated a suppressive influence^24^.

Protein synthesis is tidily controlled by the mTORC1 complex, which can be activated by increases in insulin, IGF-I and branched-chain AA concentrations such as leucine^3^. Although we initially expected a difference in mTORC1 activation between PD groups, this was not supported by current western blot analysis of mTOR phosphorylation and its downstream targets. Indeed, growth efficiency differences related to feed efficiency might occur independently of changes in mTORC1^25^. Curiously, pigs with greater growth efficiency showed a tendency for reduced Akt activation at Thr308 compared to pigs with lower feed efficiency^25^which would imply reduced protein synthesis. In the present study, Low PD gilts showed a tendency for enhanced P70S6K phosphorylation which usually indicates that the cell is signalling for increased protein synthesis and growth-related processes. Thus, contradictory results are observed in both studies when compared with the remaining literature, where the activation of mTORC1 pathway and its downstream substrates (P70S6K and 4EBP1) is rather associated with an increase in protein synthesis^4,26^. It is important to notice that, High PD gilts are in a stage of growth where maximum PD has likely already been reached and started a slow decline^12^, whereas the slow growers classified as Low PD started to reach maximum PD, thus likely contributing to variation in the observed data, and lack of statistical differences in some variables such as the activation of the mTORC1 pathway. Interestingly, Low-PD gilts showed a tendency for greater P70S6K phosphorylation in skeletal muscle, despite lower protein synthesis rates. Chronic activation of the S6K1 pathway has been linked in the literature to the development of insulin resistance through negative feedback on insulin signalling^27^. Therefore, the combination of higher circulating insulin concentrations and greater muscle P70S6K phosphorylation in Low-PD gilts may be consistent with early alterations in insulin signaling. This interpretation remains speculative and warrants further investigation.

The significant increase in ASR in High PD gilts indicates the systemic impact of muscle tissue accretion, as whole-body protein synthesis is largely driven by skeletal muscle and scales with growth rate and protein accretion in growing pigs^28.^ Whole-body protein synthesis reflects the combined activity of multiple tissues and is strongly influenced by nutrient supply, hormonal signals, and the metabolic regulation of lean tissue deposition^1.^ Increased amino acid availability and endocrine signals are known to stimulate mTOR-dependent translation initiation and muscle protein synthesis, highlighting the central role of nutrient-driven anabolic regulation during growth^29^. Consistent with this framework, High PD gilts in the present study tended to exhibit lower circulating insulin concentrations and a tendency toward higher QUICKI values, suggesting possible differences in insulin sensitivity and nutrient partitioning. Although insulin is a key stimulator of the mTORC1 pathway, its activation is also strongly regulated by amino acid availability and cellular nutrient demand. Therefore, lower circulating insulin in High PD gilts does not necessarily indicate reduced anabolic signaling, but may instead reflect greater insulin sensitivity or increased nutrient utilization associated with faster lean growth. Hormonal and nutritional factors are well recognized to redirect nutrients between protein and lipid deposition and to influence feed efficiency and lean growth in pigs^30^. However, these indices should be interpreted cautiously, as the present results cannot distinguish whether the observed endocrine differences reflect greater insulin sensitivity or increased nutrient demand associated with faster lean growth. Together, these findings support the interpretation that variation in protein deposition among gilts receiving the same diet likely reflects differences in metabolic efficiency and nutrient partitioning rather than differences in nutrient supply per se.To better understand the molecular mechanisms that may explain the observed differences between Low and High PD gilts, a transcriptomic analysis was conducted. Among the differentially expressed genes, four olfactory receptors (OR4L1, OR5D13, OR6B2, OR10R2) and one ribosomal protein (RPS15A) showed the highest fold-change between High and Low PD gilts. Additionally, eight olfactory receptors were up-regulated in High vs. Low PD gilts, representing 12% of all annotated up-regulated genes. Olfactory receptors are seven transmembrane domain G-protein coupled receptors present in olfactory sensory neurons^31^ and in several non-olfactory tissues such as the kidney, intestine, liver, heart, skeletal muscle and adipose tissues^32.^ Once activated, these receptors trigger different metabolic events including insulin secretion and fatty acid oxidation. The ORs identified in the present study have never been characterized in the skeletal muscle, however, other specific ORs are known to be involved in cell migration, cell-cell adhesion, myotube formation and muscle regeneration^32^. Moreover, it was recently reported that inactivation of the mouse OR23 gene in C2C12 myoblasts inhibits the translocation of the GLUT4 glucose transporter to the cell membrane and reduces glucose uptake^33^, thus suggesting a role in glucose homeostasis. On the other hand, activation of the mouse OR23 provoked a rise in intracellular cyclic adenosine monophosphate (cAMP), a second messenger known to have an important role in insulin sensitivity and glucose uptake in muscle tissue^34^.

The *RPS15A* gene was identified as a hub gene/protein having 11 interactions with other proteins of the PPI network. This gene encodes for a ribosomal protein that is a structural component of the 40 S subunit^32^. A protein-protein connection was observed in the PPI network between RPS15A and RPLP1, a ribosomal phosphoprotein involved in the elongation step of protein synthesis and an up-regulated gene in High vs. Low PD gilts. In addition to its role in protein translation, RPS15A participate in diverse extra-ribosomal functions. In breast cancer cells, *RPS15A* silencing reduced cell proliferation and induced apoptosis^33^ and its overexpression promoted angiogenesis in liver cancer cells^34^. The RPS15A protein has been detected in human myocytes (https://www.proteinatlas.org/) but it remains to be determined whether this protein can exert extra-ribosomal functions in skeletal muscle.

Among the annotated genes that were the most down-regulated in High *vs* Low PD gilts, *PNPO* and *SERINC2* were both reported to promote cell proliferation, migration and invasion of cancer cells^35^,^36^. The *PNPO* gene encodes for a pyridoxamine 5’-phosphate oxidase enzyme that catalyze the rate-limiting step in the synthesis of pyridoxal 5’-phosphate (PLP), the active form of vitamin B6 known to act as a cofactor for over 140 enzymes involved in various physiological processes. In skeletal muscle, vitamin B6 is essential for satellite-cell proliferation and AA metabolism^37^. The *SERINC2* gene encodes for a transmembrane protein involved in the biosynthesis of phosphatidylserine and sphingolipids^38^. Members of the SERINC proteins family (SERINC 1 to 5) are involved in membrane trafficking and SERINC1 and SERINC3 were found to colocalize with the autophagy-related protein 9 A (ATG9A)^39^, a critical component of pre-autophagosomal structures and a gene (*ATG9A*) that was also downregulated in High *vs* Low PD gilts.

The *ITGAM* gene, also known as *CD11B*, encodes for the integrin alpha M chain that is mainly expressed in macrophages, monocytes, neutrophils and dendritic cells, and participate in various pro- and anti-inflammatory processes^40^. CD11B is also a well-known cell surface marker used to identify and isolate immune cells. Therefore, variations in *ITGAM*/*CD11B* transcript levels may reflect variations in the abundance of resident immune cells in skeletal muscle of High and Low PD gilts. The presence of resident macrophages in piglets skeletal muscle was recently reported and found to represent 34.3% to 39.9% of proliferating cells^41^. Of interest, CD11B+ immune cells such as granulocytes, monocytes and macrophages are critical in satellite cell activation, myoblast proliferation and myogenic differentiation^42.^ In addition to being identified among the most down-regulated genes in High vs. Low PD gilts, ITGAM is also found in the overrepresented KEGG pathways “regulation of actin cytoskeleton” and “Rap1 signalling,” two pathways involved in cell adhesion, migration, polarity, and the polymerisation/contraction of stress fibers (Figs. 3 and 4).

The carbohydrate-binding protein AWN and STING1 genes were among the most down-regulated genes in High vs. Low PD gilts. While STING1 showed a consistent expression trend in both microarray and qPCR analysis, it did not reach significance in qPCR. As a result, its potential role in skeletal muscle growth and PD will not be addressed in the present study. The AWN protein, which is expressed in male epididymis and seminal vesicles and in female fallopian tubes, is a member of the spermadhesin family that mediates the binding of spermatozoa to oocyte^43^. The present study provides the first indication of the presence of AWN transcripts in skeletal muscle. Further work is needed to characterize its potential role in skeletal muscle.

The functional enrichment analyses identified 20 biological processes (BP) GO terms and 2 KEGG pathways. These terms can be grouped into four general categories: muscle tissue development, protein metabolism, signal transduction pathways, and response to stress. Among the 37 DEGs related to glycoprotein metabolic and protein modification processes, ten are associated with protein ubiquitination activities (ASB7, COPS7A, FBXO2, GNL3, MALT1, PSMD14, RC3H1, RNF183, SH3RF2, and WBP1L). Protein ubiquitination involves a three-step enzymatic cascade that attaches an ubiquitin protein to target proteins, leading to various cellular processes such as degradation or signal transduction^44^. Five of these genes (ASB7, MALT1, RC3H1, RNF183, and SH3RF2) are also found in the over-represented intracellular signal transduction and regulation of intracellular signal transduction GO terms. The ubiquitin-proteasome system, autophagy-lysosomal pathway, and the activation of calpains and caspases proteases are the primary proteolytic processes influencing the net balance between protein synthesis and degradation in healthy muscle^45^. Interestingly, calpain 1 (*CAPN1*) was identified as a hub gene in the PPI network and was downregulated in High vs. Low PD gilts, which may contribute to reducing skeletal muscle protein degradation.

Within the six over-represented GO terms related to muscle tissue development, five genes (MEF2D, MYH14, MYLK2, RBFOX1, and VAMP5) are shared. RBFOX1 is essential for producing a muscle-specific isoform of MEF2D, crucial in muscle cell^46^. MEF2 proteins, like MEF2D, undergo post-translational modifications such as phosphorylation and methylation, impacting their transcriptional activities. MYLK2’s phosphorylation of MEF2C enhances myogenesis^47^, yet its effect on MEF2D remains unexplored. Knockdown of MEF2D revealed its role in JAK2-like signalling and hypoxic response pathways^48^. Interestingly, two GO terms related to the hypoxic response (i.e. Response to hypoxia and response to oxygen levels) were also over-represented in the present study. In this study, MEF2D was also identified as a hub gene/protein having protein connections with the AKT1 substrate 1 (AKT1S1) gene, that encodes for an adaptor protein (PRAS40) involved in the PI3K/Akt and mTORC1 pathways^49^ and the calcineurin binding protein 1 (CABIN1) gene, known as a calcium-sensitive MEF2 transcriptional co-repressor (Han et al., 2003).

The vesicle-associated membrane protein 5 (VAMP5) gene is a member of the vesicle-associated SNAREs protein family that mediates the fusion of intracellular vesicle intermediates with target membranes. This specific isoform colocalizes with GLUT4 vesicles in rat skeletal muscle and cultured cardiomyocytes and is involved in insulin- and contraction-induced translocation of the GLUT4 glucose transporter to surface membranes^50,51^. In addition to *VAMP5*, two other genes, *BMP2* and *SIRT2*, identified within GO terms related to muscle tissue development, are also involved in cellular glucose uptake. Indeed, BMP2 is a secreted cytokine of the transforming growth factor-β family that enhances insulin-mediated glucose uptake in adipocytes^52^ and mouse C2C12 skeletal muscle cells^53^. Moreover, BMP2 have key roles in muscle growth and atrophy^54^. The *SIRT2* gene encodes for a NAD+-dependent protein deacetylase that regulates various cellular processes in metabolically active tissues. In C2C12 cells, SIRT2 protein levels are 2.7 folds higher under insulin resistance conditions, whereas SIRT2 inhibition improves insulin sensitivity and increases glucose uptake^55^.

The present study found that the regulation of actin cytoskeleton and Rap1 signalling KEGG pathways were overrepresented and interconnected. Similarly, a previous study on pig postnatal skeletal muscle growth also identified these pathways among the enriched KEGG pathways^56^. CRKL and RAPGEF1 (C3G) are hub genes identified in our study within the Rap1 signalling pathway and actin cytoskeleton regulation. CRKL transduces signals from VEGFR1 to activate Rap1 through C3G^57,58^. Rap1 activation can induce cytoskeleton rearrangements crucial for cell motility and division^58^. The downregulation of PFN1 and ITGAM in High vs. Low PD gilts was observed, indicating potential roles beyond cytoskeletal remodeling. PFN1’s involvement in actin dynamics has been linked to insulin resistance and inflammation^59^, suggesting relevance in insulin-related pathways. This is further supported by PFN1’s protective role in diet-induced insulin resistance and inflammation in mice^59^. Profilin 1 is highly expressed in adipose tissue and skeletal muscle resident macrophages but is nearly undetectable in myocytes. (The Human Protein Atlas, https://www.proteinatlas.org). Variations in PFN1 transcript levels between High and Low PD gilts may reflect differences in resident macrophages and adipocytes in skeletal muscle. This could apply similarly to integrin ITGAM, mainly expressed in immune cells such as granulocytes, monocytes, and macrophages. The crucial role of skeletal muscle resident macrophages in muscle growth was highlighted previously^60.^ In this study, a single-cell RNAseq analysis of skeletal muscle resident macrophages revealed a unique transcriptomic signature when compared with macrophages of other tissues, including a higher number of genes known to be involved in skeletal muscle growth and regeneration^60^.

Postnatal skeletal muscle growth in mammals relies on hypertrophic signals like growth hormones and mechanical stimuli, necessitating actin cytoskeleton reorganization. Among the genes that were identified within the regulation of actin cytoskeleton KEGG pathway, the Rac/Cdc42 guanine nucleotide exchange factor 6 (*ARHGEF6*) gene (also known as α-PIX) is abundantly expressed in skeletal muscle^61^. The activation of Rac1 by α-PIX is known to regulate cell-cell adhesion, migration and actin polymerization^62,63^. In skeletal muscle, Rac1 is also involved in insulin-dependent regulation of glucose uptake^64^. Rac1 can be activated by a growth factor (e.g. Insulin) via the stimulation of the Ras-PI3K-ARHGEF6 intracellular signalling pathway and by integrins such as ITGAM via focal adhesion kinase (FAK) and the CRKL adaptor protein (Fig. 5). Among Rac1 downstream effectors, the *MYLK2* (also known as skMLCK) gene was differentially expressed in High vs. Low PD gilts. The skeletal muscle myosin light chain kinase (skMLCK) phosphorylates myosin light chains and is considered as a key player in the regulation of MYH14, a myosin heavy chain that interacts with F-actin to regulate the actin cytoskeleton^65,66^. It is worth mentioning that the α-PIX protein can interact with calpain 4, the small regulatory subunit of µ-calpain (CAPN1), and active calpains were previously reported to activate Rho GTPases such as α-PIX and regulate actin filaments organization^67–69^. Although *CAPN1* was not among the list of DEGs found in the regulation of actin cytoskeleton KEGG pathway, it was identified as a hub gene/protein in the protein-protein interaction network. Among the CAPN1 interacting proteins, the ACTA1, ITGB1, RAC1, RHOA and SRC proteins are all included in the regulation of actin cytoskeleton reference pathway (https://www.kegg.jp/pathway/map04810), thus suggesting a possible role for CAPN1 in actin filaments organization.

The transcriptomic analysis highlighted molecular mechanisms governing skeletal muscle protein deposition in growing pigs, unveiling two enriched pathways (actin cytoskeleton regulation and Rap1 signalling) in High vs. Low PD gilts. Actin reorganization, vital for growth and glucose transport, underscores the significance of characterizing DEGs, particularly hub genes like *CRKL* and RAPGEF1^60,65^. The discovery of upregulated olfactory receptors presents a novel avenue for investigating non-canonical pathways in muscle growth^70.^ The discovery of ten DEGs linked to protein ubiquitination activities is also significant due to the ubiquitin-proteasome system’s role in protein synthesis and degradation balance in skeletal muscle^45^. This implies its importance in the observed differences in PD values between High and Low PD gilts. Various cell types interact within skeletal muscle, influencing its development and metabolism. Our study’s DEGs might arise from these cell types since we analyzed the whole muscle tissue, not isolated fibers. This approach revealed genes like *ITGAM* and *PFN1*, crucial for muscle growth. Additionally, DEGs encoding proteins subject to modifications like phosphorylation and ubiquitination highlight the fact that transcript levels may not reflect actual protein activities or amounts. Complementary proteomics and phosphoproteomics studies could enhance our understanding of muscle protein turnover^70^.

Overall, High PD gilts were leaner, exhibited greater muscular protein synthesis and protein synthesis efficiency, had lower postprandial insulin and C-peptide concentrations, and tended to be more insulin sensitive compared to Low PD gilts. The increased PD may reflect not only enhanced protein synthesis but also reduced protein degradation, as suggested by the downregulation of the hub gene *CAPN1* in High PD versus Low PD gilts, which may contribute to decreased skeletal muscle protein breakdown. Although postprandial glucose responses did not differ between groups, the combination of lower insulin concentrations, the trend toward higher QUICKI, and the upregulation of genes involved in insulin signaling and glucose handling, such as *VAMP5* and several genes encoding olfactory receptors, suggests subtle differences in insulin-mediated nutrient utilization rather than in glucose kinetics. Based on the overall analysis, including blood biomarkers and transcriptomic profiles, variation in PD among gilts of the same age, starting BW, and genetic line suggests the involvement of differences in nutrient utilization, insulin-related metabolic regulation, and protein turnover.

## Methods

**Fig. 8:**
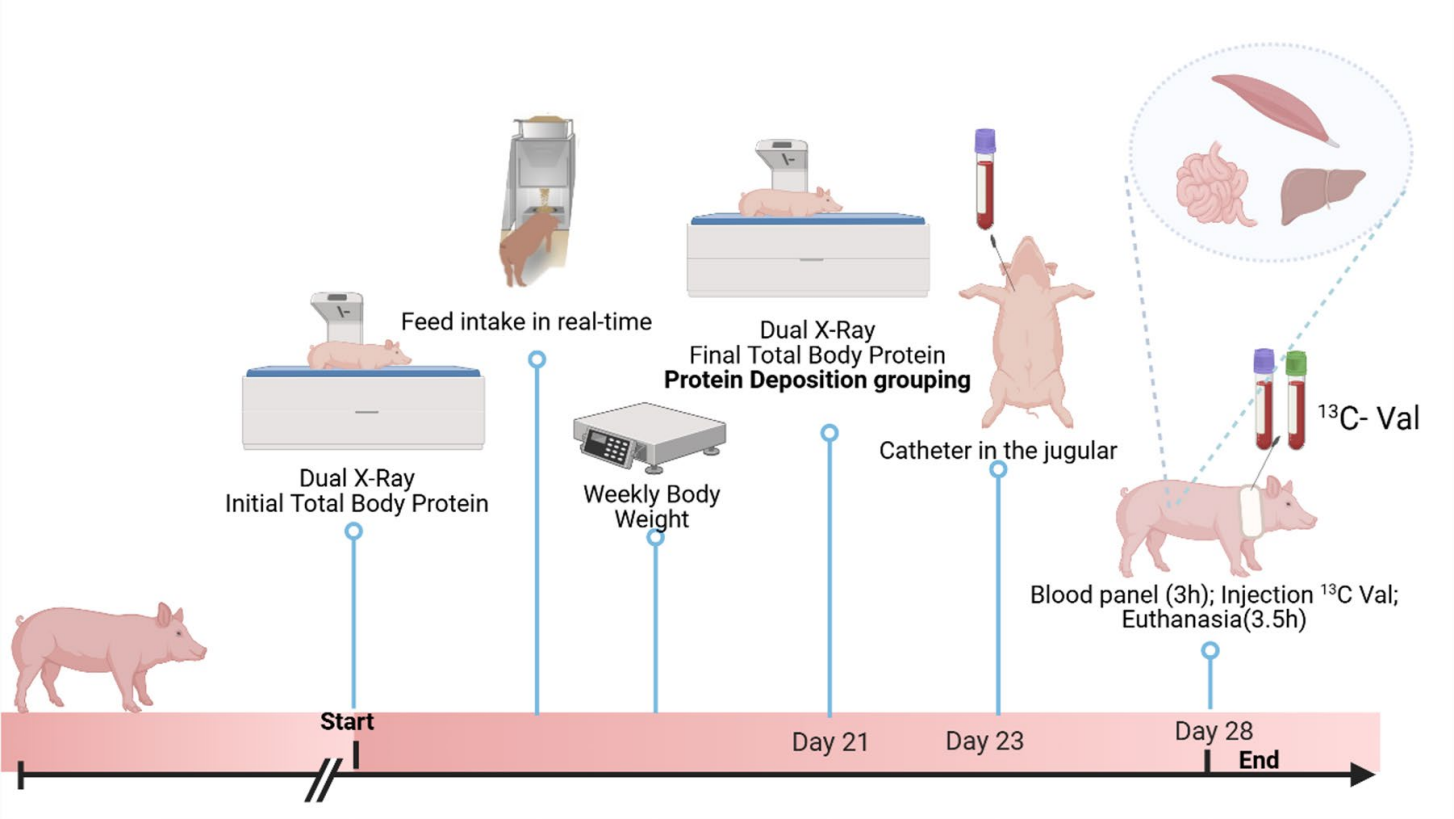
Experimental timeline for the study assessing protein synthesis and deposition in growing gilts. The experiment lasted 28 days and included dual X-ray absorptiometry (DXA) scans, real-time feed intake monitoring, weekly body weight measurements, and physiological and metabolic assessments. On Day 0, pigs were scanned by DXA to determine initial total body protein. Feed intake was recorded in real time throughout the study. On Day 21, pigs underwent a second DXA scan to determine final total body protein, and animals were subsequently classified into Low and High protein deposition groups based on their total body protein accretion between Days 0 and 21. On Day 23, a jugular catheter was implanted for isotope infusion and serial blood sampling. On Day 28, a blood panel was collected three hours before the tracer infusion, followed by an intravenous injection of L[1-^12^C]valine. After 3.5 h, pigs were euthanized, and samples of the longissimus dorsi muscle, liver, and jejunum were collected for fractional protein synthesis rate determination, hormonal assays, and transcriptomic analysis. *Created in BioRender. Remus*,* A. (2025)* https://BioRender.com/90eucob.

*Animal selection and experimental design.* Seventy growing gilts with the same genotype (AlphaGene, OLYMEL, Saint-Hyacinthe, QC, Canada) and originating from the same commercial farm entered the Agriculture and Agri-Food Canada swine complex (Sherbrooke, QC, Canada) at 33 ± 3.6 kg body weight (BW). Gilts were housed in the same room with concrete slat floors and equipped with automatic feeding stations (IVOG system, Hokofarm Group BV, Emmeloord, NE). Each pig received an individual ear tag with ID number and an electronic transponder to access the feeding stations. The adaptation period lasted seven days, during which the animals were fed ad libitum a standard commercial diet adjusted to their growing phase and had free access to water using low-pressure nipple drinkers. Then, a growing feed (Supplementary Table S4 online) formulated to maximize the growth of the most demanding pigs was fed ad libitum throughout the growing trial of 21 days. Gilts were weighted in the morning on days 1, 7, 14 and 21 of the trial, and feed intake was measured daily. On days 1 and 21, pigs were scanned to determine body protein (BP) and lipid (BL) masses^71^ using dual-energy X-ray absorptiometry (DXA; GE Lunar Prodigy Advance, Madison, WI, USA) and the total body scanning mode (Lunar encore Software version 8.10.027). For this analysis, pigs were anesthetized with sevoflurane (7%) and maintained with isoflurane (5%) throughout the scanning procedure. The timeline of the experiment is shown in Fig. 8.

*Metabolic study.* From the initial group of seventy gilts, sixteen were selected to constitute the Low (157 g/d, *n* = 8) and the High (219 g/d, *n* = 8) PD groups. They were housed in individual pens equipped with a feeder and had free access to water in a temperature-controlled room (22 °C). On day 24, using a minimally invasive procedure, they were fitted with jugular vein catheter. Pigs were given 48 h to recover from the catheter procedure and, during this time, were fed their individual average feed intake measured on days 22 and 23 divided into three meals per day. Two days before the flooding dose technique was performed, pigs were fed 90% of their average individual feed intake. They were trained to eat their meals in a short period of time (within 10 min). Pigs were fasted overnight (10 h) before proceeding with blood sampling on day 28. Fasting time was needed to determine the plasma basal AA and hormone concentration. Two blood samples were obtained 5 min apart, after which pigs were offered 300 g of a standard meal to mimic a typical meal size for pigs approximately 65 kg BW, based on natural feeding behavior. The meal was consumed within 10 min. The beginning of feeding time was considered at time 0 and blood sampling was collected at -5, -1, 10, 15, 20, 30, 40, 60, 75, 90, 120, 150 and 178 min after the meal. One gilt from the High PD group lost functionality of the catheter before labelled valine infusion, and therefore was removed from the experiment. Blood samples, collected using tubes with heparin (AA, and Val enrichment) or EDTA (glucose and hormones) as an anticoagulant, underwent centrifugation at 1,800 x g for 12 min at 4 °C within 20 min of collection. The resultant plasma was aliquoted and stored at -80 °C for AA concentrations and valine enrichment and at -20 °C for other analyses.

*L-*^*13*^*C valine injection.* At 180 min after the meal, a solution of L-^13^C valine was prepared by mixing 20% of L-[1-^13^ C] valine (Cambridge Isotope Laboratories, Inc., Tewksbury, MA, USA) with 80% of L-valine (MilliporeSigma Canada Ltd., Oakville, ON, CA) to achieve a final enrichment of 18 atom percent excess (APE) in plasma as recommended by Sève, et al.^16^. The valine was diluted in saline at 55˚C for 45 min in a vortex to reach a final concentration of 500 µmol/l. The injected dose corresponded to approximately 0.8% of body water and 3–4 times the body valine pool. The solution was prepared the morning of injection, divided into two syringes, and kept in a water bath at 37˚C until injected. Prior to injection, the solution was sterilized using a 0.22-µm filter. It was then injected via the jugular catheter at a dose of 1.05 mmol/kg of BW over approximately 5 min. The mid-injection point was considered as time zero for labeling.

Blood samples were collected before the injection (−2 min) and in the mid-injection to determine the initial values of plasma valine enrichment and hormone concentrations. After the injection, the catheter was rinsed with saline, and blood samples (6 ml) were collected at 5-min intervals from time zero until the slaughter time. At 20 min (t = 20), the pigs were euthanized, including two additional pigs for each category that did not receive ^13^C valine injection to determine the natural ^13^C abundance in different tissues. Pigs were stunned using a penetrating captive bolt device and subsequently euthanized by exsanguination, in accordance with the Canadian Council on Animal Care (CCAC) guidelines for the humane sacrifice of animals. Tissue samples (liver, 10 cm of jejunum at 100 cm from the pylorus, and 100 g of the left *longissimus* muscle at 10 cm from the midline and 6 cm from the last rib (P2)) were collected within 5 min of slaughter, cut into small cubes, and immediately frozen in liquid nitrogen. The time of freezing (last piece of tissue in liquid nitrogen) was noted and used in further calculations.

*Protein mass and content in different tissues.* Following the slaughter of the pigs, a DXA scan was conducted to determine muscle content in the body. The total carcass weight (excluding head) was recorded. The empty carcass without the head was then re-scanned with DXA to obtain the estimation of total muscle in the carcass. Lean mass estimated by DXA was converted into muscle mass using linear regression^72^. Liver and small intestine (mesentery-free) were weighed, and protein mass was obtained by multiplying the tissue weight by the analyzed protein content in the tissue (Kjeldahl Analyzer, Foss Kjeltec 8400, Palatine, IL, USA).

^13^*C valine enrichment determination.* Plasma samples (1 ml) were deproteinized using a 10% trichloroacetic acid solution and centrifuged at 1800 × g for 20 min at 4 °C. This step was repeated twice and both supernatants were combined. The plasma-free AA present in the supernatant was purified using a Dowex cation exchange resin (50 × 8,100–200 mesh, MilliporeSigma Canada Ltd., Oakville, ON, CA). Elution was carried out with 4 M NH_4_OH, followed by drying with N and recovery in water.

Tissue samples (1 g) were ground in a 0.2 mol/l perchloric acid solution. The protein precipitate was collected after centrifugation at 1800 × g for 20 min at 4 °C, washed three times with the perchloric acid solution, homogenized with 10 ml of 0.3 mol/l NaOH, and gently stirred for 1 h at 37 °C. A 1 ml aliquot was taken for protein concentration measurement. Proteins were precipitated once again with 2 mol/l perchloric acid, followed by centrifugation at 1800 × g for 20 min at 4 °C. The supernatant was used to determine total RNA concentrations. After two additional washes with 0.2 mol/l perchloric acid, the protein precipitate was hydrolyzed in 12 mol/l HCl for 48 h at 110 °C. A hydrolysis duration of 48 h was used, as this condition was previously validated in our laboratory to maximize the release of free valine. The resulting solution was filtered, dried, and suspended in water.

Gas chromatography-mass spectrometry (GC-MS) was used to measure the AA concentrations by isotopic dilution after addition of labelled internal standard to the samples^73^, and plasma valine enrichments (Agilent Technologies 7890B GC coupled to 5977 A MS detector, Mississauga, ON, Canada), using N-(tert-butyldimethyl) AA derivative. Protein-bound valine enrichments in tissues were determined using the GC-C-IRMS (HP 6890 N GC coupled to an IsoPrime MS, GV Instruments, Manchester, UK) after derivatization with ethyl chloroformate. The fractional synthesis rate (FSR, %/day), representing the percentage of protein mass synthesized per day, was calculated using the following equation:

$${\mathrm{FSR~}}\left( {{{\% }}/{\mathrm{day}}} \right) = {\mathrm{~}}100 \times \left( {\frac{{{\mathrm{Ebound}}}}{{time~ \times Efree}}} \right)$$

where Ebound is the isotopic enrichment of protein-bound valine, and Efree is the isotopic enrichment of free valine in each tissue (muscle, intestine, liver), both corrected for natural abundance measured on the plasma. Finally, time is the elapsed time between the injection of L-[1-^13^ C] valine and the animals’ sacrifice plus the average time at completion of tissue freezing in liquid nitrogen.

The absolute synthesis rate ASR (g/day) was obtained by multiplying the total protein mass (pool size) by the FSR:

$$\:\mathrm{A}\mathrm{S}\mathrm{R}\:\left(\mathrm{g}/\mathrm{d}\mathrm{a}\mathrm{y}\right)=\:\mathrm{F}\mathrm{S}\mathrm{R}\times\:\mathrm{t}\mathrm{i}\mathrm{s}\mathrm{s}\mathrm{u}\mathrm{e}\:\mathrm{p}\mathrm{o}\mathrm{o}\mathrm{l}\:\mathrm{s}\mathrm{i}\mathrm{z}\mathrm{e}$$

*Total RNA concentration in skeletal muscle.* A solution containing 0.3 M NaOH and 20% perchloric acid (PA) was first prepared by mixing 900 ml of NaOH with 275 ml of PA. The longissimus dorsi muscle supernatant was diluted 5 times with this solution. The diluted sample was measured at two wavelengths: 260 nm and 232 nm. A blank was prepared using only the NaOH/PA solution. The RNA concentration (µg/ml) was calculated using the formula: RNA (µg/ml) = 10.53 × (3.4 × A260–1.44 × A232), where A represented the absorbance.

The ribosomal capacity for protein synthesis (Cs) was used as an indicator of cellular potential for protein synthesis. It was calculated as the ratio of RNA to protein (mg/g) in each tissue:

$$\:Cs=\frac{mg\:RNA}{g\:protein}$$

Additionally, the efficiency of protein synthesis (K_RNA_) was evaluated as the amount of protein synthesized per ribosomal RNA (g protein synthesized/day/mg RNA) using the following equation:

$$\:{K}_{\mathrm{R}\mathrm{N}\mathrm{A}}=\left(\frac{FSR\:\times\:10}{Cs}\right)$$

The Cs represents the potential for protein synthesis and is expressed as the RNA-to-protein ratio (mg RNA/g protein), reflecting the cellular machinery available for translation^16,74^. Conversely, the K_RNA_ reflects the amount of protein synthesized per unit of ribosomal RNA (g protein synthesized/day/mg RNA) and indicates how effectively the translational machinery is being used^16,74^.

*Hormones and indexes.* Plasma glucose concentration was determined using an enzymatic colorimetric assay (No 997–03001, Wako Life Sciences, Mountain View, CA, USA). Insulin concentration was assessed using a commercial porcine insulin RIA kit (#PI-12 K; EMD Millipore Corporation, Saint Charles, MO, USA), and C-peptide concentration was measured using an ELISA kit (C-peptide porcine No 10-1256-01, Mercodia Inc, Winston-Salem, NC, USA). Insulin sensitivity in the basal state was estimated using the quantitative insulin sensitivity check index (QUICKI)^75^:

$$\:QUICKI\:=\frac{1}{\left[ln\left(fasting\:plasma\:insulin\right)+ln\left(fasting\:plasma\:glucose\right)\right]}$$

The Homeostasis model assessment (HOMA) was used to estimate insulin resistance (HOMA-IR) and β-cell function (HOMA- %B) at basal conditions as previously described^76^:

$$\:HOMA-IR=\left(\frac{fasting\:plasma\:insulin\times\:fasting\:plasma\:glucose}{\mathrm{22,5}}\right)$$

$$\:HOMA-\%B=\frac{20\:\times\:fasting\:plasma\:insulin}{fasting\:plasma\:glucose-3.5}$$

Plasma IGF-1 concentrations were analyzed in duplicate with a standard curve in triplicate using the IGF-I RIA kit (reference number 22-IGF-R21 Mediagnost, Reutlingen, Germany). Blood samples were collected using lavender-topped tubes with EDTA as an anticoagulant and underwent centrifugation at 1,800 x g for 12 min at 4 °C within 20 min of collection. Plasma aliquots were stored at −20 °C until analysis. Assay buffers, antibodies, tracers and standards were prepared as recommended by the manufacturer and stored at −10 °C and −20 °C after reconstitution. Intra- and inter-assay variability were assessed.

*RNA isolation and evaluation of integrity.* Total RNA was isolated from the *longissimus* muscle (30 mg) of gilts with High (*n* = 6) and Low (*n* = 6) PD values using the RNeasy Mini Kit (Qiagen, Toronto, ON, Canada) with the optional on-column DNase I digestion step. Extracted RNA was quantified using the NanoDrop Spectrophotometer ND-1000 (Thermo Fisher Scientific, Wilmington, DE, USA) and its integrity assessed with the 2100 Bioanalyzer system (Agilent Technologies, Santa Clare, CA, USA). RNA integrity numbers (RIN) of isolated RNA samples ranged from 7.5 to 9.1, thus meeting quality criteria for transcriptomic analyses (Schroeder et al., 2006).

*Microarray hybridization and data analyses.* The cDNA synthesis, fragmentation, labeling and microarray hybridizations were performed at the “Centre d’expertise et de services Génome Québec” (https://cesgq.com/, Montreal, QC, Canada). For each sample, the GeneChip^®^ WT Terminal Labeling Kit was used to synthesize the sense-strand cDNA from 100 ng of total RNA, followed by fragmentation and labeling of cDNA probes according to the manufacturer’s instructions (Thermo Fisher Scientific). Labeled cDNA probes (3.5 µg) were then hybridized to Affymetrix GeneChip^®^ Porcine Gene 1.0 ST Arrays (#901977, Thermo Fisher Scientific) that were incubated for 17 h, at 45 °C and 60 rpm in a Genechip^®^ Hybridization oven 640 (Thermo Fisher Scientific). This porcine specific array includes 394,580 probes for a total of 19,212 genes. Each array was then washed in a GeneChips^®^ Fluidics Station 450 (Thermo Fisher Scientific) using the GeneChip Hybridization Wash and Stain kit and finally scanned on a GeneChip^®^ scanner 3,000 (Thermo Fisher Scientific). The .CEL files were then loaded onto R and processed by the Canadian Centre for Computational Genomics (C3G; Montréal, QC, Canada). The oligo package within the R environment was used for raw data visualization and intensities normalization of .CEL files^77^. Identification and clustering of differentially expressed genes (DEGs) between High and Low PD gilts was carried out with the R package *limma* (linear models for microarray analysis; Ritchie et al., 2015). Genes with a cut-off threshold > 1.15 for fold change (FC) and a Benjamini–Hochberg false discovery rate (FDR)–adjusted*P*-value ≤ 0.05 were considered as differentially expressed and kept for further analyses. The complete list of DEGs is available in Supplementary Table S1 online.

*Gene ontology and KEGG pathways enrichment analyses.* The Database for Annotation, Visualization and Integrated Discovery (DAVID)^78^ knowledgebase (version 2023q1, https://david.ncifcrf.gov/home.jsp) was used to carry out functional enrichment analyses of DEGs. The uploaded gene list (official gene symbols) included 67 up- and 102 down-regulated annotated unique genes (Supplementary Table S2 online). Enriched Gene Ontology (GO) terms for biological process (BP) were identified using the *Sus scrofa* reference gene list and the GOTERM_BP_ALL annotation category. The minimum number of genes was set at 5 and the expression analysis systemic explorer (EASE) score threshold set at 0.05 (modified Fisher Exact *P*-value).

Biological pathways enrichment analyses were performed with the Kyoto Encyclopedia of Genes and Genomes (KEGG) database (release 104.1)^79^ from DAVID, using an EASE, which corresponds to a modified Fisher’s Exact test based on a hypergeometric distribution. Pathways were considered enriched at an EASE score threshold of 0.10, a fold enrichment score (FES) > 1.5, a minimum 5 genes per pathway, and the *Sus scrofa* gene annotation list used as background.

*Protein-protein interactions network analysis.* The PPI network was generated using the list of DEGs identified based on FDR-adjusted P-values. In order to visualize protein-protein interactions (PPI) among DEGs, a PPI network was generated with the Search Tool for Retrieval of Interacting Genes/Proteins (STRING) interactome database^80^, using the NetworkAnalyst 3.0 visual analytic platform (https://www.networkanalyst.ca/)^77^. The network was built by uploading the complete list of up- and down-regulated DEGs (total of 169) in High vs. Low PD gilts (Supplementary Table S2 online) and using the generic PPI and the minimum network tool that keeps seed genes (e.g. uploaded genes) and non-seed genes to maintain connections. The STRING interactome database was selected using the first-order building option and a high confidence score cutoff of 900. The resulting network includes nodes (proteins encoded by genes) and edges that represent interactions between proteins. The node explorer table associated with the network provides two topological measures: the node degree that corresponds to the number of connections that a node has with other nodes and betweenness centrality (BC) defined as the number of shortest paths going through a node. Proteins with the highest degree and BC values were considered as core proteins (hub genes) with important functions in skeletal muscle PD. The figure quality was enhanced by reproducing the network using BioRender (https://www.biorender.com/), the original figure is presented in Supplementary Figure S2.

*Quantitative RT-PCR analyses.* For validation of microarray results, the mRNA abundance of 20 randomly selected DEGs was analyzed in the skeletal muscle of gilts with High (*n* = 6) and Low (*n* = 6) PD values. To confirm identity, sequencing of each amplified gene was first conducted (Centre d’expertise et de services Génome Québec). Total RNA isolation was performed as described above. For each sample, the cDNA synthesis was performed with 1 µg of total RNA, oligo (dt) 20 primers and the SuperScript^®^ IV Reverse Transcriptase, according to the manufacturer’s instructions (Thermo Fisher Scientific). The cDNA was then diluted 1:15 for qPCR analyses using an ABI 7500 Fast Real-Time PCR System (PE Applied Biosystems, Foster City, CA, USA). The PCR reactions (in triplicates), amplification conditions and melting curve analyses were as previously reported^81^. For each gene, 300 nM of forward and reverse primers were used for the amplification reactions (Supplementary Table S3 online). Three reference genes (RG) were also amplified: hypoxanthine phosphoribosyltransferase 1 (*HPRT1*), peptidylpropyl isomerase A (*PPIA*) and DNA topoisomerase II beta (*TOP2B*). For each gene and RG, a standard curve was generated in duplicate as previously described^82^. The relative mRNA abundance was then calculated with the relative standard curve method^83^ and by dividing the relative quantity units of selected genes by those of RGs. Among the three RGs, PPIA was identified as the most stable gene by the NormFinder algorithm from the Excel-Tools-Add-ins^84^, and was therefore used for mRNA abundance normalization. The mean values from triplicates were finally used for statistical analyses. Amplification efficiencies (E) of each gene are presented in Supplementary Table S3 online and were calculated with the following equation: E = 10 ^[− 1/slope]^ and conversion of E into a percentage ((E-1) x 100).

*Western Blot analyses.* Western blot analyses (Supplementary Figure S1) were performed to determine if there are differences in the mammalian target of rapamycin (mTOR) phosphorylation (as a proxy for mTORC1 activation) in skeletal muscle of gilts having High (*n* = 4) or Low (*n* = 4) PD values. Selected gilts were those showing more evident clustering (Fig. 2b) between these two groups. Protein extraction was carried-out with the T-PER tissue protein extraction reagent (Thermo Fisher Scientific) in the presence of phenylmethylsulfonyl fluoride (PMSF, 1X) and the Halt protease inhibitor cocktail (1X) to inhibit proteases (Thermo Fisher Scientific). Protein concentration was then determined with the Bio-Rad DC protein assay (BIO-RAD, Mississauga, ON, Canada). Ten (10) µg of extracted proteins was loaded in each well of a prolyacrylamide gel (SDS-PAGE; 6% for mTOR (289 kDA), 10% for p70S6 kinase (70 kDa) and 15% for 4E-BP1 (15 kDa)) and separated by electrophoresis (1 h at 150 Volts) using the Mini Gel Tank electrophoresis chamber system (Thermo Fisher Scientific). Protein transfer on PVDF membranes (BIO-RAD) was performed with the same chamber system (1.5 h at 100 Volts). Membranes were blocked with 5% skimmed milk in TBST (20 mM Tris-HCl, pH 7.5, 150 mM NaCl, 0.1% Tween-20) for 2 h at room temperature and incubated overnight at 4 °C with primary antibodies against mTOR (7C10) (1:1000; rabbit mAb #2983S), phospho-mTOR at Ser2448 (1:500, rabbit polyclonal #2971S), p70 S6 kinase (1:1000, rabbit mAb #34475S), phospho-p70 S6 kinase at Thr389 (1:500, rabbit polyclonal #9205S), 4E-BP1 (1:1000, rabbit polyclonal #9452S) and phospho-4E-BP1 at Thr70 (1:1000, rabbit polyclonal #9455S). Membranes were then incubated for 1 h with a goat anti-rabbit IgG secondary antibody (1:5000, HRP-linked #7074S). These antibodies were purchased from Cell Signaling Technology (Beverly, MA, USA). Membranes were also incubated with anti α-tubulin primary antibodies (1:10000, mouse monoclonal #T5168) and goat anti-mouse IgG secondary antibodies (1:10000, HRP-linked ab97040) that were used as loading control (Abcam, Branford, CT, USA). For these antibodies, a single band was observed at the expected molecular weight (Western blot raw images available in Supplementary Figure S1). Protein detection was performed with the Amersham ECL Select Western Blotting Reagent (Sigma-Aldrich, St-Louis, MO, USA) following the manufacturer’s instructions. The Fusion FX4 imaging system (Montreal Biotech, QC, Canada) was used to detect and quantify chemiluminescent signals. For each target (mTOR, 4EBP1, and P70S6K), total and phosphorylated forms were analyzed on the same membrane following sequential stripping and reprobing, while different proteins were analyzed on separate membranes. α-tubulin was used as a loading control and probed on the same membranes as the target proteins. Phosphorylated proteins were first normalized to their corresponding total protein abundance to obtain phosphorylation ratios. These ratios were subsequently normalized to α-tubulin to account for potential differences in protein loading between lanes.

### Statistical analyses 

Growth performance, protein synthesis measurements and correlation analysis were performed using RStudio (version 4.2.3; R Foundation for Statistical Computing, Vienna, Austria). Data were analyzed as a complete randomized design using the lmerTest package^76^, considering the individual pig as the experimental unit. Due to experimental constraints, pigs were randomly distributed over 3 days to receive the flooding-dose injection. Thus, the main fixed effect was the PD group, and the ^13^C-valine injection day was considered a random effect. The assumption of normal distribution of the error, homogeneity of variances based on the Bartlett test, influential values and outliers presence were checked using the performance package^77^. Observations were excluded from the analysis based on the detection outlier using Cook’s distance method with a threshold of 0.5 based on the whole model. The uncertainty in the estimate of the means of the data was expressed as the maximum standard error (MSE). Relative mRNA abundance was normalized using reference genes selected with the NormFinder algorithm, and the most stable gene combinations were used to calculate normalized expression values prior to statistical analysis. To analyze the effect of PD (High vs. Low) on the mRNA abundance of selected genes (qPCR validation) and on the activation of mTOR (Western blots), a one-way analysis of variance (ANOVA) with homogeneous variances was conducted using the MIXED procedure of SAS (2012; SAS Institute Inc., Cary, NC, USA). An ANOVA with heterogeneous variances was also performed for some specific genes (*ACO2*, *MEF2D*, *RHBDD2* and *VAMP5*). A Shapiro-Wilk test was applied to verify the normality of the residuals, and a Wilcoxon test was performed as a parametric confirmatory test. Statistical significance was set at *P* ≤ 0.05 and tendencies at *P* ≤ 0.10. Given the biological variability inherent to animal experiments and the limited number of experimental units per treatment, using*0.05 < P ≤ 0.10 a*s an indicator of a tendency provides a balanced interpretation of results, reducing the risk of Type II errors while maintaining appropriate statistical rigor^85^.

## Supplementary Information

Below is the link to the electronic supplementary material.

Supplementary Material 1

Supplementary Material 2

## Data Availability

The datasets generated during the current study belong to His Majesty the King in Right of Canada, as represented by the Minister of Agriculture and Agri-Food Canada, and are not publicly available. Data can be obtained from the authors (aline.remus@agr.gc.ca) upon reasonable request and with the permission of the representative of His Majesty the King in Right of Canada.

## References

[1] Sarri, L., Balcells, J., Seradj, A. R. & de la Fuente, G. Protein turnover in pigs: A review of interacting factors. *J. Anim. Physiol. Anim. Nutr.* **108**, 451–469. 10.1111/jpn.13906 (2024). 10.1111/jpn.13906 37975299

[2] Davis, T. A., Burrin, D. G., Fiorotto, M. L. & Nguyen, H. V. Protein synthesis in skeletal muscle and jejunum is more responsive to feeding in 7-than in 26 day-old pigs. *Am. J. Physiol. Endocrinol. Metab.*. **270**, E802–E809 (1996). 10.1152/ajpendo.1996.270.5.E802 8967468

[3] Davis, T. A. et al. Stimulation of protein synthesis by both insulin and amino acids is unique to skeletal muscle in neonatal pigs. *Am. J. Physiol. Endocrinol. Metab.*. **282**, E880–E890 (2002). 10.1152/ajpendo.00517.2001 11882508

[4] Suryawan, A. et al. Activation by insulin and amino acids of signaling components leading to translation initiation in skeletal muscle of neonatal pigs is developmentally regulated. *Am. J. Physiol. Endocrinol. Metab.*. **293**, E1597–E1605. 10.1152/ajpendo.00307.2007 (2007). 10.1152/ajpendo.00307.2007 PMC2714663 17878222

[5] Salgado, H. H. et al. Insulin sensitivity is associated with the observed variation of de novo lipid synthesis and body composition in finishing pigs. *Sci. Rep.* **12**, 14586 (2022). 36028540 10.1038/s41598-022-18799-0 PMC9418310

[6] Poore, K. R. & Fowden, A. L. Insulin sensitivity in juvenile and adult Large White pigs of low and high birthweight. *Diabetologia* **47**, 340–348. 10.1007/s00125-003-1305-3 (2004). 14722651 10.1007/s00125-003-1305-3

[7] Sandri, M. et al. Signalling pathways regulating muscle mass in ageing skeletal muscle. The role of the IGF1-Akt-mTOR-FoxO pathway. *Biogerontology* **14**, 303–323 (2013). 23686362 10.1007/s10522-013-9432-9

[8] Cruzen, S. M. et al. Evidence of decreased muscle protein turnover in gilts selected for low residual feed intake1. *J. Anim. Sci.* **91**, 4007–4016. 10.2527/jas.2013-6413 (1986). 10.2527/jas.2013-6413 23739790

[9] Hauschild, L., Lovatto, P. A., Pomar, J. & Pomar, C. Development of sustainable precision farming systems for swine: Estimating real-time individual amino acid requirements in growing-finishing pigs. *J. Anim. Sci.* **90**, 2255–2263. 10.2527/jas.2011-4252 (2012). 22287679 10.2527/jas.2011-4252

[10] Remus, A., del Castillo, J. & Pomar, C. Improving the estimation of amino acid requirements to maximize nitrogen retention in precision feeding for growing-finishing pigs. *Animal*, (2020). 10.1017/S1751731120000798 10.1017/S1751731120000798 32319362

[11] Remus, A., Hauschild, L., Corrent, E., Létourneau-Montminy, M. P. & Pomar, C. Pigs receiving daily tailored diets using precision-feeding techniques have different threonine requirements than pigs fed in conventional phase-feeding systems. *J. Anim. Sci. Biotechnol.* **10**, 16. 10.1186/s40104-019-0328-7 (2019). 30834113 10.1186/s40104-019-0328-7 PMC6385469

[12] Remus, A., Hauschild, L., Methot, S. & Pomar, C. Precision livestock farming: Real-time estimation of daily protein deposition in growing–finishing pigs. *Animal* **14**, s360–s370. 10.1017/S1751731120001469 (2020). 32583758 10.1017/S1751731120001469

[13] Reeds, J. P. & Lobley, E. G. Protein synthesis: Are there real species differences? *Proc. Nutr. Soc.* **39**, 43–52. 10.1079/PNS19800007 (1980). 10.1079/pns19800007 6988836

[14] Mnilk, B., Harris, C. I. & Fuller, M. F. Lysine utilization by growing pigs: Simultaneous measurement of protein accretion and lysine oxidation. *Br. J. Nutr.* **75**, 57–67. 10.1079/BJN19960110 (1996). 8785191 10.1079/bjn19960110

[15] Blumenberg, M. in *Transcriptome Analysis* (ed Miroslav Blumenberg)IntechOpen, (2019).

[16] Sève, B. et al. Recombinant porcine somatotropin and dietary protein enhance protein synthesis in growing pigs. *J. Nutr.* **123**, 529–540. 10.1093/jn/123.3.529 (1993). 7681874 10.1093/jn/123.3.529

[17] Remus, A. et al. O68 Gilts with different protein deposition do not differ in mRNA abundance of genes related to nutrient transport by the small intestine. *Anim. - Sci. Proc.* **13**, 328–329. 10.1016/j.anscip.2022.07.078 (2022).

[18] Remus, A. et al. O52 Variability in protein deposition is linked to changes in transcript abundance of genes involved in mTOR signaling pathways in growing pigs. *Anim. Sci. Proc.* 13, 307–308, (2022). 10.1016/j.anscip.2022.07.062

[19] Hawkes, C. P. & Grimberg, A. Insulin-like growth factor-i is a marker for the nutritional state. *Pediatr. Endocrinol. Rev.* **13**, 499–511 (2015). 26841638 PMC5576178

[20] Tuvdendorj, D. et al. Amino acid availability regulates the effect of hyperinsulinemia on skin protein metabolism in pigs *. *J. Biol. Chem.* **290**, 17776–17783. 10.1074/jbc.M114.636100 (2015). 26032410 10.1074/jbc.M114.636100 PMC4505026

[21] Waterlow, J. C., Garlick, P. J. & Mill Ward, D. *Protein turnover in mammalian tissues and in the whole body*. (1978).

[22] Sève, B. & Ponter, A. A. Nutrient-hormone signals regulating muscle protein turnover in pigs. *Proc. Nutr. Soc.* 56, 565–580, (1997). 10.1079/PNS19970058 10.1079/pns19970058 9264108

[23] Nam, S. et al. Effect of obesity on total and free insulin-like growth factor (IGF)-1, and their relationship to IGF-binding protein (BP)-1, IGFBP-2, IGFBP-3, insulin, and growth hormone. *Int. J. Obes.* **21**, 355–359 (1997). 10.1038/sj.ijo.0800412 9152736

[24] Ahlman, B. et al. Insulin’s effect on synthesis rates of liver proteins: A swine model comparing various precursors of protein synthesis. *Diabetes* **50**, 947–954. 10.2337/diabetes.50.5.947 (2001). 11334437 10.2337/diabetes.50.5.947

[25] Cruzen, S. M. et al. Evidence of decreased muscle protein turnover in gilts selected for low residual feed intake1. *J. Anim. Sci.* **91**, 4007–4016. 10.2527/jas.2013-6413 (2013). 23739790 10.2527/jas.2013-6413

[26] Bond, P. Regulation of mTORC1 by growth factors, energy status, amino acids and mechanical stimuli at a glance. *J. Int. Soc. Sports Nutr.* **13**, 8. 10.1186/s12970-016-0118-y (2016). 26937223 10.1186/s12970-016-0118-y PMC4774173

[27] Jia, G., Aroor, A. R., Martinez-Lemus, L. A., Sowers, J. R. & Overnutrition mTOR signaling, and cardiovascular diseases. *Am. J. Physiol. Regul. Integr. Comp. Physiol.* **307**, R1198–R1206. 10.1152/ajpregu.00262.2014 (2014). 10.1152/ajpregu.00262.2014 PMC4233289 25253086

[28] Reeds, P. J., Cadenhead, A., Fuller, M. F., Lobley, G. E. & McDonald, J. D. Protein turnover in growing pigs. Effects of age and food intake. *Br. J. Nutr.* **43**, 445–455 (1980). 7417390 10.1079/bjn19800112

[29] Suryawan, A. & Davis, T. A. Amino acid- and insulin-induced activation of mTORC1 in Neonatal piglet skeletal muscle involves sestrin2-GATOR2, Rag A/C-mTOR, and RHEB-mTOR complex formation. *J. Nutr.* **148**, 825–833. 10.1093/jn/nxy044 (2018). 29796625 10.1093/jn/nxy044 PMC6669959

[30] Gondret, F. et al. Dietary energy sources affect the partition of body lipids and the hierarchy of energy metabolic pathways in growing pigs differing in feed efficiency1,2. *J. Anim. Sci.* **92**, 4865–4877. 10.2527/jas.2014-7995 (2014). 25253805 10.2527/jas.2014-7995

[31] Buck, L. & Axel, R. A novel multigene family may encode odorant receptors: A molecular basis for odor recognition. *Cell* **65**, 175–187. 10.1016/0092-8674(91)90418-X (1991). 1840504 10.1016/0092-8674(91)90418-x

[32] Lee, S. J., Depoortere, I. & Hatt, H. Therapeutic potential of ectopic olfactory and taste receptors. *Nat. Rev. Drug Discovery*. **18**, 116–138. 10.1038/s41573-018-0002-3 (2019). 30504792 10.1038/s41573-018-0002-3

[33] Kang, W., Zhang, K., Tong, T. & Park, T. Improved glucose intolerance through a distinct mouse olfactory receptor 23-induced signaling pathway mediating glucose uptake in myotubes and adipocytes. *Mol. Nutr. Food Res.* **64**, 1901329. 10.1002/mnfr.201901329 (2020). 10.1002/mnfr.201901329 32918394

[34] Yang, H. & Yang, L. Targeting cAMP/PKA pathway for glycemic control and type 2 diabetes therapy. *J. Mol. Endocrinol.* **57**, R93–R108. 10.1530/jme-15-0316 (2016). 27194812 10.1530/JME-15-0316

[35] Ren, W., Guan, W., Zhang, J., Wang, F. & Xu, G. Pyridoxine 5’-phosphate oxidase is correlated with human breast invasive ductal carcinoma development. *Aging (Albany NY)*. **11**, 2151–2176. 10.18632/aging.101908 (2019). 30982780 10.18632/aging.101908 PMC6503878

[36] Zeng, Y. et al. SERINC2–knockdown inhibits proliferation, migration and invasion in lung adenocarcinoma. *Oncol. Lett.* **16**, 5916–5922. 10.3892/ol.2018.9403 (2018). 30405754 10.3892/ol.2018.9403 PMC6202524

[37] Komaru, T., Yanaka, N. & Kumrungsee, T. Satellite cells exhibit decreased numbers and impaired functions on single myofibers isolated from vitamin B6-deficient mice. *Nutrients* **13**, 4531 (2021). 34960083 10.3390/nu13124531 PMC8705767

[38] Inuzuka, M., Hayakawa, M. & Ingi, T. Serinc, an Activity-regulated protein family, incorporates serine into membrane lipid synthesis*. *J. Biol. Chem.* **280**, 35776–35783. 10.1074/jbc.M505712200 (2005). 16120614 10.1074/jbc.M505712200

[39] Davies, A. K. et al. AP-4 vesicles contribute to spatial control of autophagy via RUSC-dependent peripheral delivery of ATG9A. *Nat. Commun.* **9**, 3958. 10.1038/s41467-018-06172-7 (2018). 30262884 10.1038/s41467-018-06172-7 PMC6160451

[40] Khan, S. Q., Khan, I. & Gupta, V. CD11b Activity modulates pathogenesis of lupus nephritis. *Front. Med.* **5**10.3389/fmed.2018.00052 (2018). 10.3389/fmed.2018.00052 PMC5862812 29600248

[41] Albrecht, E., Zhao, Y., Sciascia, Q. L., Metges, C. C. & Maak, S. Identification and quantification of proliferating cells in skeletal muscle of glutamine supplemented low- and normal-birth-weight piglets. *Cells* **12**, 580 (2023). 36831247 10.3390/cells12040580 PMC9953894

[42] Bentzinger, C. F., Wang, Y. X., Dumont, N. A. & Rudnicki, M. A. Cellular dynamics in the muscle satellite cell niche. *EMBO Rep.* **14**, 1062–1072. 10.1038/embor.2013.182 (2013). 24232182 10.1038/embor.2013.182 PMC3849491

[43] Haase, B. et al. Evolution of the spermadhesin gene family. *Gene* **352**, 20–29. 10.1016/j.gene.2005.04.015 (2005). 15922517 10.1016/j.gene.2005.04.015

[44] Swatek, K. N. & Komander, D. Ubiquitin modifications. *Cell Res.* **26**, 399–422. 10.1038/cr.2016.39 (2016). 27012465 10.1038/cr.2016.39 PMC4822133

[45] Yin, L. et al. Skeletal muscle atrophy: From mechanisms to treatments. *Pharmacol. Res.* **172**, 105807. 10.1016/j.phrs.2021.105807 (2021). 34389456 10.1016/j.phrs.2021.105807

[46] Runfola, V., Sebastian, S., Dilworth, F. J. & Gabellini, D. Rbfox proteins regulate tissue-specific alternative splicing of Mef2D required for muscle differentiation. *J. Cell Sci.* **128**, 631–637. 10.1242/jcs.161059 (2015). 25609712 10.1242/jcs.161059 PMC4357529

[47] Al Madhoun, A. S. et al. Skeletal myosin light chain kinase regulates skeletal myogenesis by phosphorylation of MEF2C. *EMBO J.* **30**, 2477–2489. 10.1038/emboj.2011.153 (2011). 21556048 10.1038/emboj.2011.153 PMC3116284

[48] Estrella, N. L. et al. MEF2 transcription factors regulate distinct gene programs in mammalian skeletal muscle differentiation. *J. Biol. Chem.* **290**, 1256–1268. 10.1074/jbc.M114.589838 (2015). 25416778 10.1074/jbc.M114.589838 PMC4294490

[49] Lv, D., Guo, L., Zhang, T. & Huang, L. PRAS40 signaling in tumor. *Oncotarget* **8** (2017). 10.18632/oncotarget.17299 PMC5620322 28978182

[50] Rose, A. J., Jeppesen, J., Kiens, B. & Richter, E. A. Effects of contraction on localization of GLUT4 and v-SNARE isoforms in rat skeletal muscle. *Am. J. Physiol. Regul. Integr. Comp. Physiol.* **297**, R1228–R1237. 10.1152/ajpregu.00258.2009 (2009). 10.1152/ajpregu.00258.2009 19675279

[51] Schwenk, R. W. et al. Requirement for distinct vesicle-associated membrane proteins in insulin- and AMP-activated protein kinase (AMPK)-induced translocation of GLUT4 and CD36 in cultured cardiomyocytes. *Diabetologia* **53**, 2209–2219. 10.1007/s00125-010-1832-7 (2010). 20582536 10.1007/s00125-010-1832-7 PMC2931635

[52] Schreiber, I. et al. BMPs as new insulin sensitizers: Enhanced glucose uptake in mature 3T3-L1 adipocytes via PPARγ and GLUT4 upregulation. *Sci. Rep.* **7**, 17192. 10.1038/s41598-017-17595-5 (2017). 29222456 10.1038/s41598-017-17595-5 PMC5722815

[53] Kohler, Z. M. et al. Tilorone increases glucose uptake in vivo and in skeletal muscle cells by enhancing Akt2/AS160 signaling and glucose transporter levels. *J. Cell. Physiol.* **238**, 1080–1094. 10.1002/jcp.30998 (2023). 37012691 10.1002/jcp.30998

[54] Sartori, R. et al. BMP signaling controls muscle mass. *Nat. Genet.* **45**, 1309–1318. 10.1038/ng.2772 (2013). 24076600 10.1038/ng.2772

[55] Arora, A. & Dey, C. S. SIRT2 negatively regulates insulin resistance in C2C12 skeletal muscle cells. *Biochim. et Biophys. Acta (BBA) - Mol. Basis Disease*. **1842**, 1372–1378. 10.1016/j.bbadis.2014.04.027 (2014). 10.1016/j.bbadis.2014.04.027 24793418

[56] Wang, Y. et al. Dynamic transcriptome profiles of postnatal porcine skeletal muscle growth and development. *BMC Genomic Data*. **22**10.1186/s12863-021-00984-1 (2021). 10.1186/s12863-021-00984-1 PMC8419915 34488628

[57] Rodríguez-Blázquez, A. et al. Crk proteins activate the Rap1 guanine nucleotide exchange factor C3G by segregated adaptor-dependent and -independent mechanisms. *Cell. Communication Signal.* **21**10.1186/s12964-023-01042-2 (2023). 10.1186/s12964-023-01042-2 PMC9896810 36737758

[58] Jaśkiewicz, A., Pająk, B. & Orzechowski, A. The many faces of rap1 GTPase. *Int. J. Mol. Sci.* **19**, 2848 (2018). 30241315 10.3390/ijms19102848 PMC6212855

[59] Romeo, G. R., Pae, M., Eberlé, D., Lee, J. & Shoelson, S. E. Profilin-1 haploinsufficiency protects against obesity-associated glucose intolerance and preserves adipose tissue immune homeostasis. *Diabetes* **62**, 3718–3726. 10.2337/db13-0050 (2013). 23884883 10.2337/db13-0050 PMC3806603

[60] Wang, X. et al. Heterogeneous origins and functions of mouse skeletal muscle-resident macrophages. *Proc. Natl. Acad. Sci.* **117**, 20729–20740. 10.1073/pnas.1915950117 (2020). 32796104 10.1073/pnas.1915950117 PMC7456122

[61] Manser, E. et al. PAK kinases are directly coupled to the PIX family of nucleotide exchange factors. *Mol. Cell.* **1**, 183–192. 10.1016/s1097-2765(00)80019-2 (1998). 9659915 10.1016/s1097-2765(00)80019-2

[62] Zhou, W., Li, X. & Premont, R. T. Expanding functions of GIT Arf GTPase-activating proteins, PIX Rho guanine nucleotide exchange factors and GIT–PIX complexes. *J. Cell Sci.* **129**, 1963–1974. 10.1242/jcs.179465 (2016). 27182061 10.1242/jcs.179465 PMC6518221

[63] Bosco, E. E., Mulloy, J. C. & Zheng, Y. Rac1 GTPase: A Rac of All Trades. *Cell. Mol. Life Sci.* **66**, 370. 10.1007/s00018-008-8552-x (2008). 10.1007/s00018-008-8552-x PMC6669905 19151919

[64] Møller, L. L. V., Klip, A. & Sylow, L. Rho GTPases—Emerging regulators of glucose homeostasis and metabolic health. *Cells* **8**, 434 (2019). 31075957 10.3390/cells8050434 PMC6562660

[65] Chinthalapudi, K., Heissler, S. M., Preller, M., Sellers, J. R. & Manstein, D. J. Mechanistic insights into the active site and allosteric communication pathways in human nonmuscle myosin-2C. *eLife* **6**, e32742. 10.7554/eLife.32742 (2017). 29256864 10.7554/eLife.32742 PMC5749951

[66] Wang, K., Okada, H. & Bi, E. Comparative analysis of the roles of non-muscle myosin-iis in cytokinesis in budding yeast, fission yeast, and mammalian cells. *Front. Cell. Dev. Biol.*. **8**10.3389/fcell.2020.593400 (2020). 10.3389/fcell.2020.593400 PMC7710916 33330476

[67] Sato, K. & Kawashima, S. Calpain function in the modulation of signal transduction molecules. *Biol. Chem.* **382**, 743–752. 10.1515/bchm.2001.382.5.743 (2001). 11517927 10.1515/BC.2001.090

[68] Rosenberger, G., Gal, A. & Kutsche, K. AlphaPIX associates with calpain 4, the small subunit of calpain, and has a dual role in integrin-mediated cell spreading. *J. Biol. Chem.* **280**, 6879–6889. 10.1074/jbc.M412119200 (2005). 15611136 10.1074/jbc.M412119200

[69] Kulkarni, S., Saido, T. C., Suzuki, K. & Fox, J. E. B. Calpain Mediates Integrin-induced Signaling at a Point Upstream of Rho Family Members*. *J. Biol. Chem.* **274**, 21265–21275. 10.1074/jbc.274.30.21265 (1999). 10409684 10.1074/jbc.274.30.21265

[70] Draicchio, F. et al. Involvement of the extracellular matrix and integrin signalling proteins in skeletal muscle glucose uptake. *J. Physiol.* **600**, 4393–4408. 10.1113/JP283039 (2022). 36054466 10.1113/JP283039 PMC9826115

[71] Pomar, C. & Rivest, J. in *Proceedings of the 46th Annual Conference of the Canadian Society of Animal Science, Lethbridge, Alberta.*

[72] Marcoux, M., Faucitano, L. & Pomar, C. The accuracy of predicting carcass composition of three different pig genetic lines by dual-energy X-ray absorptiometry. *Meat Sci.* **70**, 655–663. 10.1016/j.meatsci.2005.02.015 (2005). 22063893 10.1016/j.meatsci.2005.02.015

[73] Calder, A. G., Garden, K. E., Anderson, S. E. & Lobley, G. E. Quantitation of blood and plasma amino acids using isotope dilution electron impact gas chromatography/mass spectrometry with U-(13)C amino acids as internal standards. *Rapid Commun. Mass. Spectrom.* **13**, 2080–2083. 10.1002/(sici)1097-0231(19991115)13:21<2080::Aid-rcm755>3.0.Co;2-o (1999). 10523763 10.1002/(SICI)1097-0231(19991115)13:21<2080::AID-RCM755>3.0.CO;2-O

[74] Hamard, A. & Sève, B. Le Floc’h, N. A moderate threonine deficiency differently affects protein metabolism in tissues of early-weaned piglets. *Comp. Biochem. Physiol. A: Mol. Integr. Physiol.* **152**, 491–497. 10.1016/j.cbpa.2008.12.002 (2009). 19095073 10.1016/j.cbpa.2008.12.002

[75] Katz, A. et al. Quantitative insulin sensitivity check index: A simple, accurate method for assessing insulin sensitivity in humans. *J. Clin. Endocrinol. Metabolism*. **85**, 2402–2410. 10.1210/jcem.85.7.6661 (2000). 10.1210/jcem.85.7.6661 10902785

[76] Matthews, D. R. et al. Homeostasis model assessment: Insulin resistance and β-cell function from fasting plasma glucose and insulin concentrations in man. *Diabetologia* **28**, 412–419. 10.1007/BF00280883 (1985). 3899825 10.1007/BF00280883

[77] Carvalho, B. S. & Irizarry, R. A. A framework for oligonucleotide microarray preprocessing. *Bioinformatics* **26**, 2363–2367. 10.1093/bioinformatics/btq431 (2010). 20688976 10.1093/bioinformatics/btq431 PMC2944196

[78] Sherman, B. T. et al. DAVID: A web server for functional enrichment analysis and functional annotation of gene lists (2021 update). *Nucleic Acids Res.* **50**, W216–W221. 10.1093/nar/gkac194 (2022). 35325185 10.1093/nar/gkac194 PMC9252805

[79] Kanehisa, M., Furumichi, M., Sato, Y., Kawashima, M. & Ishiguro-Watanabe, M. KEGG for taxonomy-based analysis of pathways and genomes. *Nucleic Acids Res.* **51**, D587–D592. 10.1093/nar/gkac963 (2022). 10.1093/nar/gkac963 PMC9825424 36300620

[80] Szklarczyk, D. et al. STRING v11: Protein–protein association networks with increased coverage, supporting functional discovery in genome-wide experimental datasets. *Nucleic Acids Res.* **47**, D607–D613. 10.1093/nar/gky1131 (2018). 10.1093/nar/gky1131 PMC6323986 30476243

[81] Palin, M. F., Caron, A. & Farmer, C. Effects of sustained hyperprolactinemia in late gestation on the mammary parenchymal tissue transcriptome of gilts. *BMC Genom.* **24**, 40. 10.1186/s12864-023-09136-4 (2023). 10.1186/s12864-023-09136-4 PMC9875420 36694114

[82] Labrecque, B. et al. Molecular characterization and expression analysis of the porcine paraoxonase 3 (PON3) gene. *Gene* **443**, 110–120. 10.1016/j.gene.2009.04.026 (2009). 19426787 10.1016/j.gene.2009.04.026

[83] Biosystems, A. Applied Biosystems Foster City, CA, (1997).

[84] Vandesompele, J. et al. Accurate normalization of real-time quantitative RT-PCR data by geometric averaging of multiple internal control genes. *Genome Biol.* **3**10.1186/gb-2002-3-7-research0034 (2002). research0034.0031. 10.1186/gb-2002-3-7-research0034 PMC126239 12184808

[85] Thiese, M. S., Ronna, B. & Ott, U. P value interpretations and considerations. *J. Thorac. Dis.* **8**, E928–e931. 10.21037/jtd.2016.08.16 (2016). 27747028 10.21037/jtd.2016.08.16 PMC5059270

