# Supplementary material for: Insights into protein synthesis dynamics of gilts from the same genetic background and age differing in protein deposition
Source: Sci Rep. 2026 May 15;16:22170. doi: 10.1038/s41598-026-52194-3 (PMC13369171; doi:10.1038/s41598-026-52194-3)
Supplement: Supplementary file 2 — Supplementary Material 2 [file 41598_2026_52194_MOESM2_ESM.pdf]

Supplementary material

Insights into protein synthesis dynamics of gilts from the same genetic background and age differing in protein deposition

Aline Remus[1 *]; Marie-France Palin[1]; Hélène Lapierre[1], Nathalie Le Floc'h[2], Jaap van Milgen[2], Candido Pomar[1]

[1]Sherbrooke Research and Development Centre, Agriculture and Agri-Food Canada, Sherbrooke, Québec, Canada, J1M 0C8;

[2]INRAe, UMR1348 Pegase, Saint-Gilles, France; and Agrocampus-Ouest, UMR1348 Pegase, Rennes, France;

[*]aline.remus@agr.gc.ca

	Count	Official gene symbols[1]
Up-regulated genes (High vs Low)	67	AKAP7, APCDD1L, ART3, BMP2, CDCA4, CDH16, CHN1, CLEC19A, CPNE8, CRYGS, CTF2, DLEU7, ESPL1, FAM78B, FANCD2OS, FBXO2, FGFR2IIIC, GDF3, GK2, GLIPR1L1, GNGT2, GNL3, GRIP1, GSDMC, HOXB9, HOXD13, HS6ST3, HYAL4, LAGE3, LDLRAD1, LRRC75B, LYRM2, MAP3K6, MMP27, NCS1, OR10R2, OR10S1, OR2B8P, OR4L1, OR4N5, OR5B2, OR5D13, OR6B2, OTP, PDSS1, PRDM12, PRR18, PSMD14, PWWP2B, PYROXD2, RAD9B, RELL2, RNF183, RPLP1, RPS15A, SDCCAG8, SDHD, SERHL2, SEZ6L2, SNRPG, TCEAL4, TIGD7, TMEM132C, TRMT9B, TTLL8, WARS2, ZGLP1
Down-regulated genes (High vs Low)	102	ACO2, ACP2, AGAP1, AKT1S1, ALAS1, ALG12, AP2A1, ARHGEF6, ASB7, ATG9A, ATP2A3, ATXN2L, AWN, AXL, BAHD1, BAZ2A, BCAT2, BCL3, BICRA, Btbd11, CABIN1, CAPN1, CC2D1B, CCDC6, COPS7A, COX10, CRKL, CS, CYHR1, DENND4B, DUSP3, EIF2AK1, EPDR1, EXTL3, FOXO4, GATAD2B, GTPBP2, HCK, HOXC6, HSPB6, INPP5D, IPO13, ITGAM, LARP1, LMBR1L, LRP1, MALT1, MAN2A2, MED15, MEF2D, Mrps18a, MYH14, MYLK2, NAA80, NAV2, NDRG1, NTNG1, PCMTD1, PDCB4, PDPR, PFN1, PHF12, PLEKHM2, PNPO, PPM1D, RAPGEF1, RBFOX1, RC3H1, RFX5, RHBDD2, RNF10, SAP130, SCAMP3, SERINC2, SF3A1, SH3BGRL3, SH3RF2, SIMC1, SIRT2, SKIV2L, SLC19A1, SLC38A3, SLC41A3, SLCO5A1, SORBS1, SP2, SPEG, SRPRA, STING1, STRN4, TET2, TMEM259, TMOD1, TRIM66, TRIM8, TUBA8, VAMP5, VEGFB, VPS28, WBP1L, XK, ZBTB40

Supplementary Table S2. List of up- and down-regulated genes in the longissimus muscle of gilts with High vs Low protein deposition values. [1]These genes were included in a unique gene list that was uploaded in the DAVID bioinformatics resource database for functional annotation clustering of differentially expressed genes and in the NetworkAnalyst 3.0 visual analytic platform for protein-protein interactions network analysis.

Genes[1]	Primer sequences (5'-3')[2]	GenBank accession no.	Product size (bp)	Amplification efficiency (%)[3]
**Selected genes**				
ACO2	(F)GTGTACCACTGACCACATCTC	NM_213954	90	96.44
	(R)GCACCGATGAGCAAGTTATTG			
ATG9A	(F)CTGGGAGATCCACTCCTTCTA	NM_001190275	122	93.37
	(R)TGCAGATCTGGTGCTCTTTC			
BCAT2	(F)GCTGATCCTCCGCTTCTATAAC	XM_003127278	134	106.03
	(R)GACTGGATGCTACAGGTCATT			
BMP2	(F)CCCGGCGCTTCTTCTTTAAT	NM_001195399	124	105.82
	(R)CGGTGATGGAAACTGCTACTG			
CDCA4	(F)CGTCGCTGGATCAGATATTGG	XM_005656478	101	99.96
	(R)GGCGTCCAGGTCGTAGTA			
FOXO4	(F)TGGAGTGTGACATGGATAAC	XM_003135172	120	100.18
	(R)CTCATCTCTGAAGCAAGGAA			
MAP3K6	(F)GAGACCTTCACAGGAACACTAC	XM_013988796	107	105.81
	(R)ATGACAGTGCAGCCCAAT			
MEF2D	(F)GACAAGGTGCTGCTCAAGTA	XM_021089672	83	100.73
	(R)TTCCTCAGGGTCTCGATGAT			
MMP27	(F)GGTCGGTGTCCTCGTTATTT	XM_013979325	122	99.13
	(R)GTTCCATCCTTGGTCCAGTT			
NCS1	(F)GCCCGAATGTCTCCTCTTTAG	XM_021070198	109	97.08
	(R)GAGCCGAGAAACTGAGAGATG			
PSMD14	(F)GGAACAGGTGTCAGTGTAGAAG	XM_003359536	137	99.49
	(R)CCAGAAAGCCAACAACCAAAG			
RAPGEF1	(F)GTCCGTGAAGATTCCAGAGAAG	XM_005654593	124	101.76
	(R)CACAGCACTGGTGGACATAA			
RHBDD2	(F)GAGTTTCTAGGGAAGTGGTCTC	XM_021086307	102	103.31
	(R)CACATCGAGGTGTTTGGTAAAG			
SDHD	(F)TGGAGGCTCAGTGTTCTTTG	NM_001097516	107	98.28
	(R)GTATGTCGGTCCTGGAGAAATG			
SERINC2	(F)TCTGTGTCTGCGTCTCCATA	NM_001244148	140	97.50
	(R)CTGGTCAGGGACATTGGATAAG			
STING1	(F)TGGACTGGCCTGGTCTTATTA	NM_001142838	95	95.01
	(R)CGTTCTTGTGGCGCTGATTA			
TCEAL4	(F)GGCTCAGTGTCTCAAGGAATAC	XM_003135283	108	106.72
	(R)TTCACCTCATCCTCCACTCT			
VAMP5	(F)GGATGAGGTGACGGAAATCAT	XM_005655212	103	98.58
	(R)CATGTCCAGGAGTTGGTCTG			
VPS28	(F)AACAAGCCGGAGCTGTATG	XM_001927458	132	97.57
	(R)GTCCTTGATGTACGCCTTCTC			
ZGLP1	(F)CCCAACTCAGGAAGGGTAAG	XM_013987414	113	101.52
	(R)CTCAGGACTCAGGCTCTTTC			
**Reference genes**				
HPRT1	(F)GACCAGACTTTGTTGGATTTGAAA	NM_001032376	94	100.27
	(R)CAAACATGATTCAAGTCCCTGAAG			
PPIA	(F)GCACTGGTGGCAAGTCCAT	NM_214353	71	99.81
	(R)AGGACCCGTATGCTTCAGGA			
TOP2B	(F)AAGACGGCACCAAAAGGTAAAG	NM_001258386	120	99.03
	(R)CTTCGGTTTCTTGCTTGTTGTTT			

Supplementary Table S3. Primer sequences used for real-time PCR amplification of selected genes. [1]*ACO2*, aconitase 2; *ATG9A*, autophagy related 9A; *BCAT2*, branched chain amino acid transaminase 2; *BMP2*, bone morphogenetic protein 2; *CDCA4*, cell division cycle associated 4; *FOXO4*, forkhead box O4; *HPRT1*, hypoxanthine phosphoribosyltransferase 1; *MAP3K6*, mitogen-activated protein kinase kinase kinase 6; *MEF2D*, myocyte enhancer factor 2D; *MMP27*, matrix metallopeptidase 27; *NCS1*, neural calcium sensor 1; *PPIA*, peptidylpropyl isomerase A; *PSMD14*, proteasome 26S subunit, non-ATPase 14; *RAPGEF1*, rap guanine nucleotide exchange factor 1; *RHBDD2*, rhomboid domain containing 2; *SDHD*, succinate dehydrogenase complex subunit D; *SERINC2*, serine incorporator 2; *STING1*, stimulator of interferon response CGAMP interactor 1; *TCEAL4*, transcription elongation factor A like 4; *TOP2B*, DNA topoisomerase II beta; *VAMP5*, vesicle associated membrane protein 5; *VPS28*, VPS28 subunit of ESCRT-I; *ZGLP1*, zinc finger GATA like protein 1. [2]Forward (F) and reverse (R) primers. [3]Amplification efficiency (E) was calculated with $E=10^{[-1/slope]}$.

Ingredient name	Amount (g/kg)
Corn	600.46
Soybean meal	184
Wheat	171
Limestone	13.7
Fat surface	9
Monocalcium phosphate	5.38
L-lysine Hcl	4.75
Salt (39%)	4.6
Micromineral and vitamin premix	2
L-threonine	1.9
Dl-methionine (99%)	1.45
L-valine	0.84
L-tryptophan	0.62
Choline chloride (65%)	0.2
Phytate (Phytase activity minimum 5000)	0.1
**Chemical composition**	**%**
Dry matter	87.77
Crude protein	16.08
Fat (ether)	3.49
Crude fiber	2.62
SID lysine	1.02
SID methionine	0.37
SID methionine + cysteine	0.61
SID threonine	0.66
SID tryptophan	0.22
SID arginine	0.90
SID histidine	0.36
SID isoleucine	0.55
SID leucine	1.20
SID phenylalanine	0.71
SID valine	0.71
Net energy, MJ/kg	10.43
Calcium	0.70
Phosphorous total	0.43
Phosphorous available	0.32

Supplementary Table S4. Ingredient and chemical composition (as-fed basis) of the experimental feed.

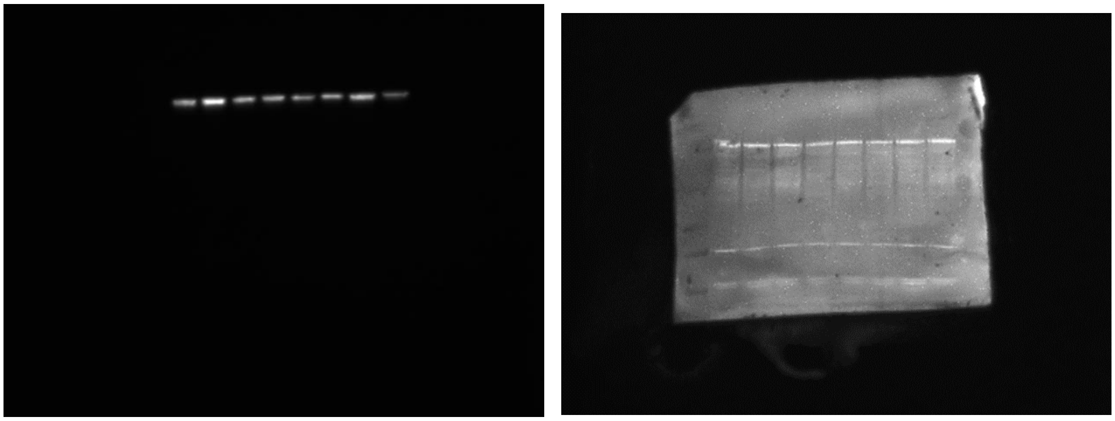

mTOR

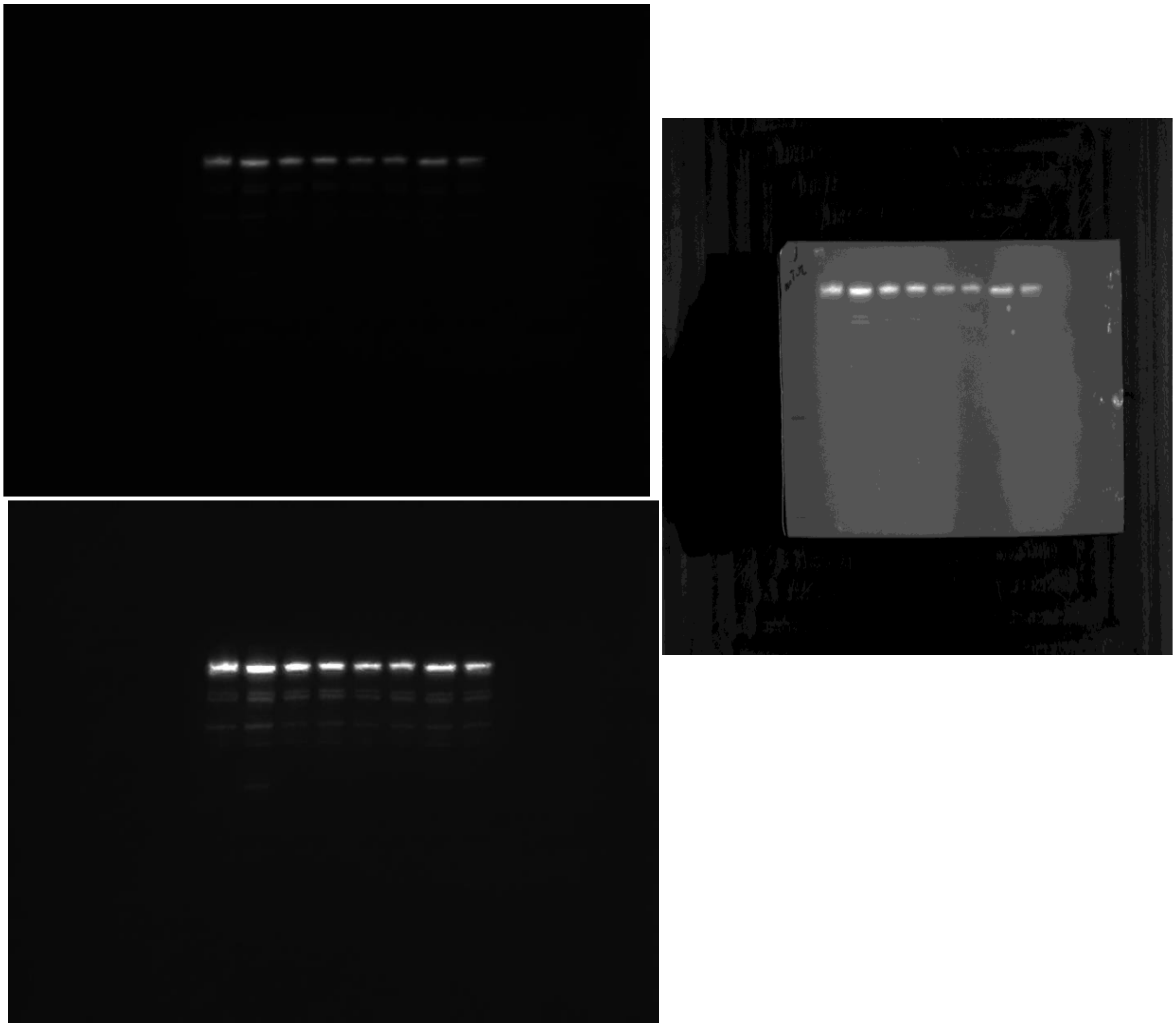

p-mTOR

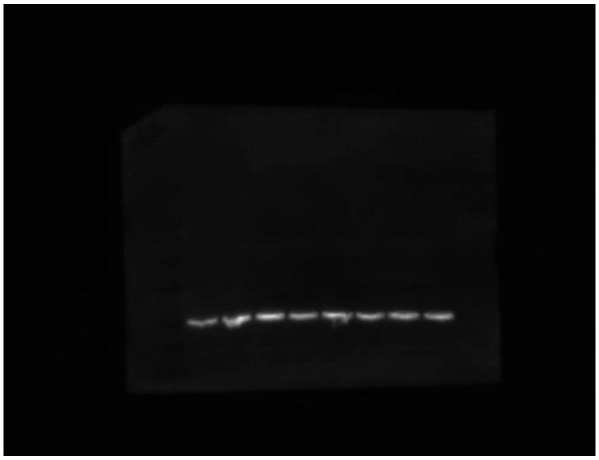

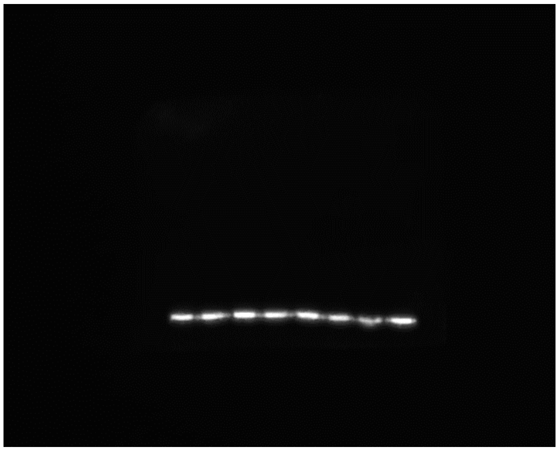

α-Tubulin

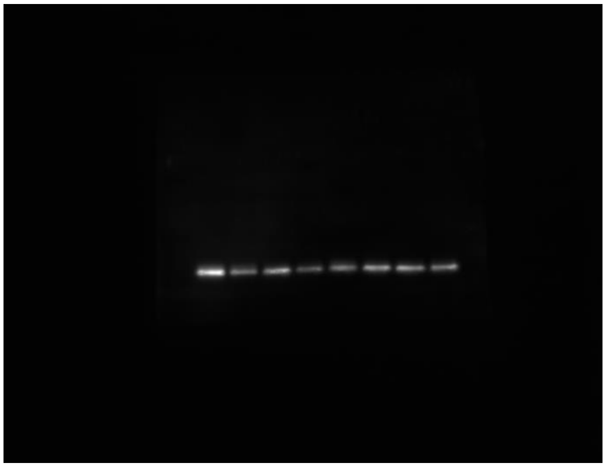

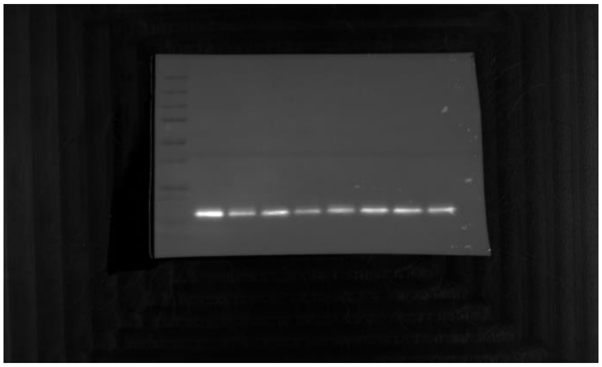

4EBP1

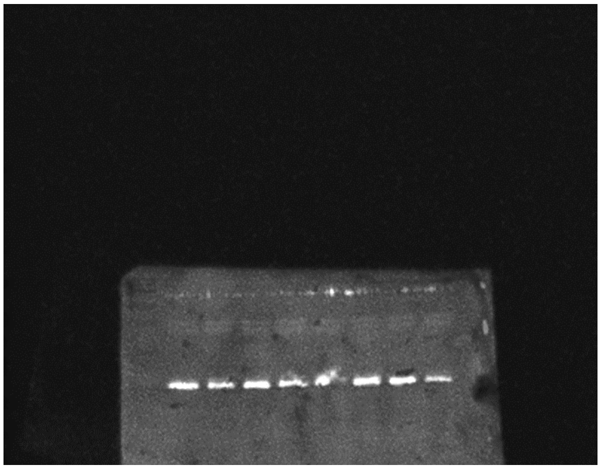

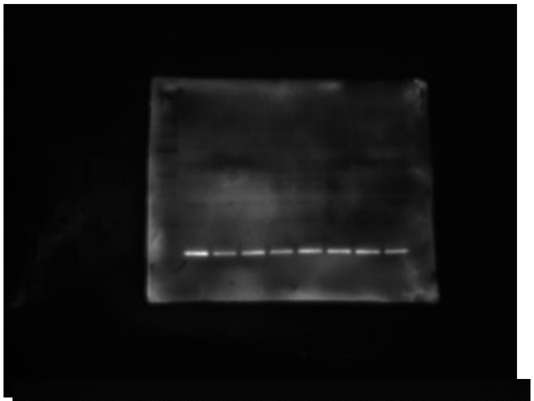

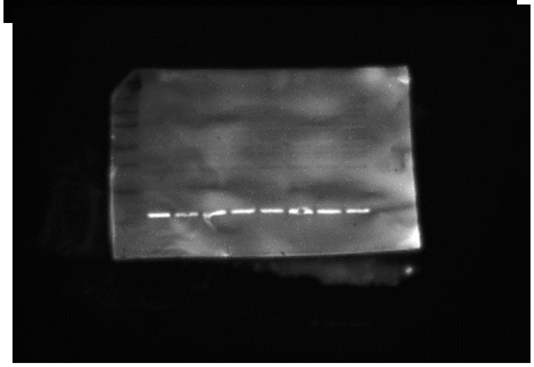

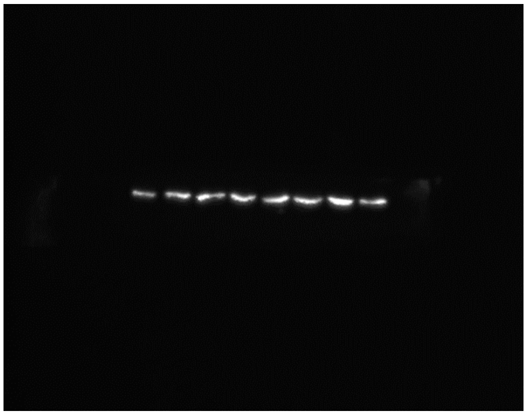

p-4EBP1

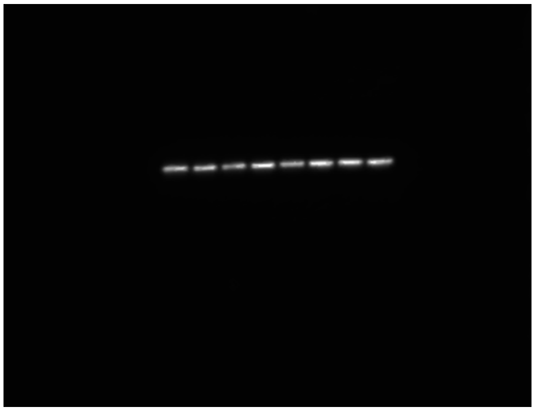

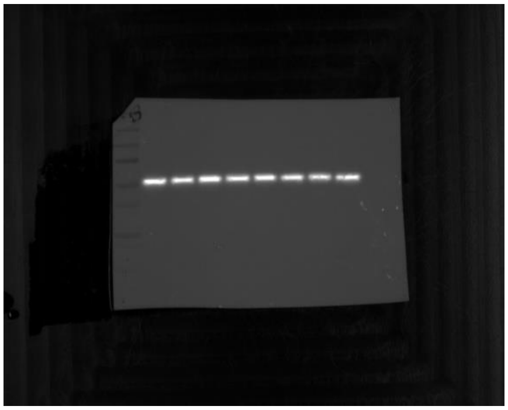

α-Tubulin

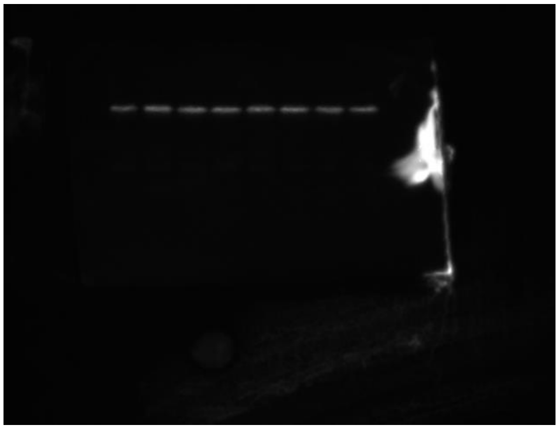

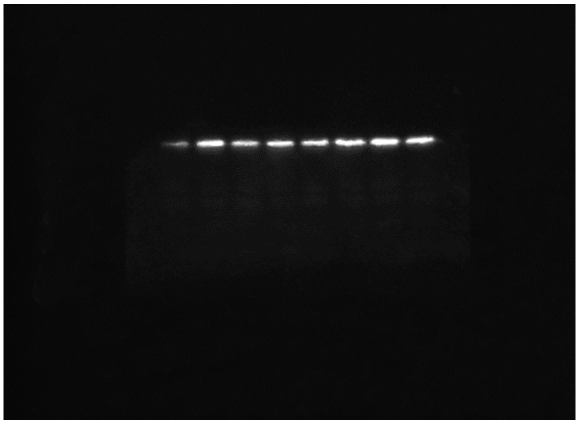

P70 S6
[illegible]

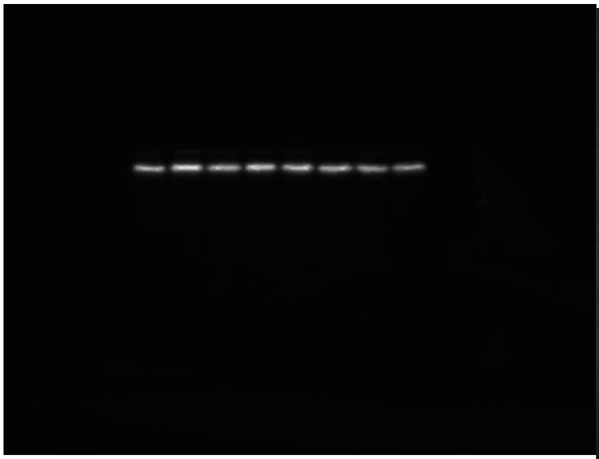

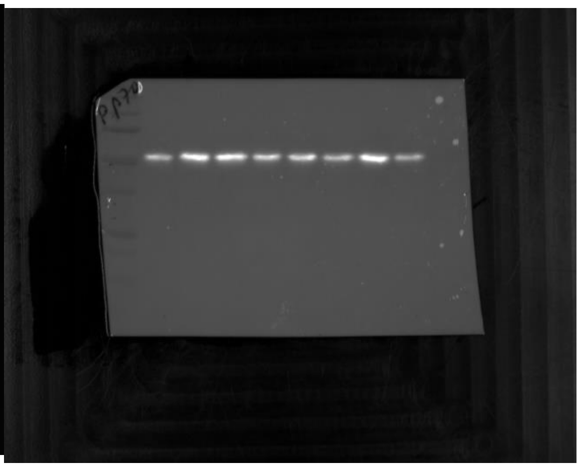

p-P70 S6
kinase

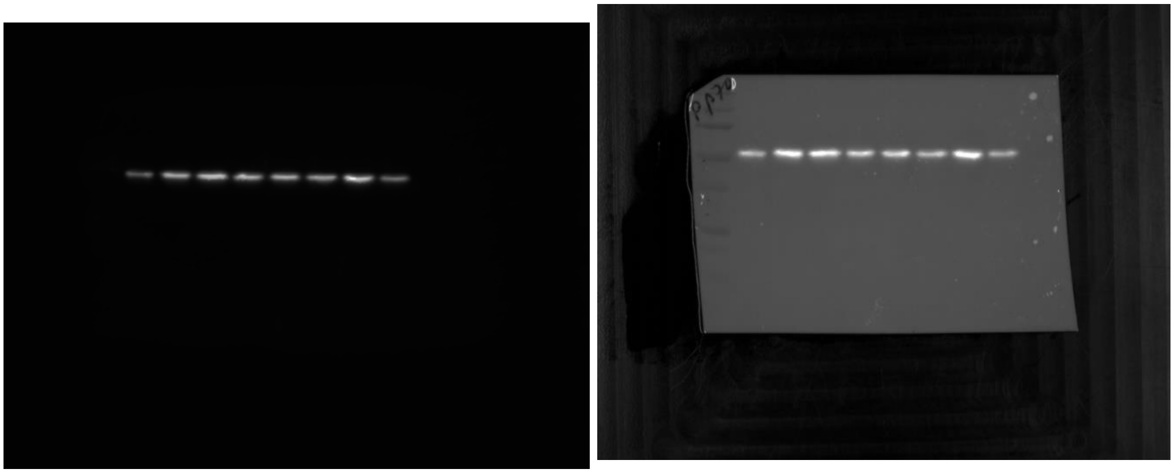

α-Tubulin

Supplementary Figure S1. Full-length, uncropped Western blot images, in sequenced from above to bellow are the mTOR, p-mTOR, α-Tubulin, 4EBP1, p-4EBP1, α-Tubulin, P70 S6 kinase, p-P70 S6 kinase, and α-Tubulin in the longissimus muscle of gilts with High and Low protein deposition values. Each target protein was analyzed on separate membranes, probed sequentially following membrane stripping. α-Tubulin was used as a loading control and was probed on the same membranes as target proteins. Bands are labeled by protein and ordered as presented in Figure 7. The following primary antibodies were used: mTOR (289 kDa, rabbit mAb #2983S), phospho-mTOR at Ser2448 (rabbit polyclonal #2971S), p70 S6 kinase (70 kDa, rabbit mAb #34475S), phospho-p70 S6 kinase at Thr389 (rabbit polyclonal #9205S), 4EBP1 (15 kDa, rabbit polyclonal #9452S), and phospho-4EBP1 at Thr70 (rabbit polyclonal #9455S), all from Cell Signaling Technology. The loading control α-tubulin (50 kDa) was detected using a mouse monoclonal antibody (#T5168, Abcam). Secondary antibodies used were HRP-linked goat anti-rabbit IgG (#7074S) and goat anti-mouse IgG (#ab97040). Images presented in Figure 7 were uniformly adjusted for brightness and contrast to improve clarity; the original, unprocessed (non-enhanced) full-length images are shown here. Molecular weight markers were included to confirm band identities.

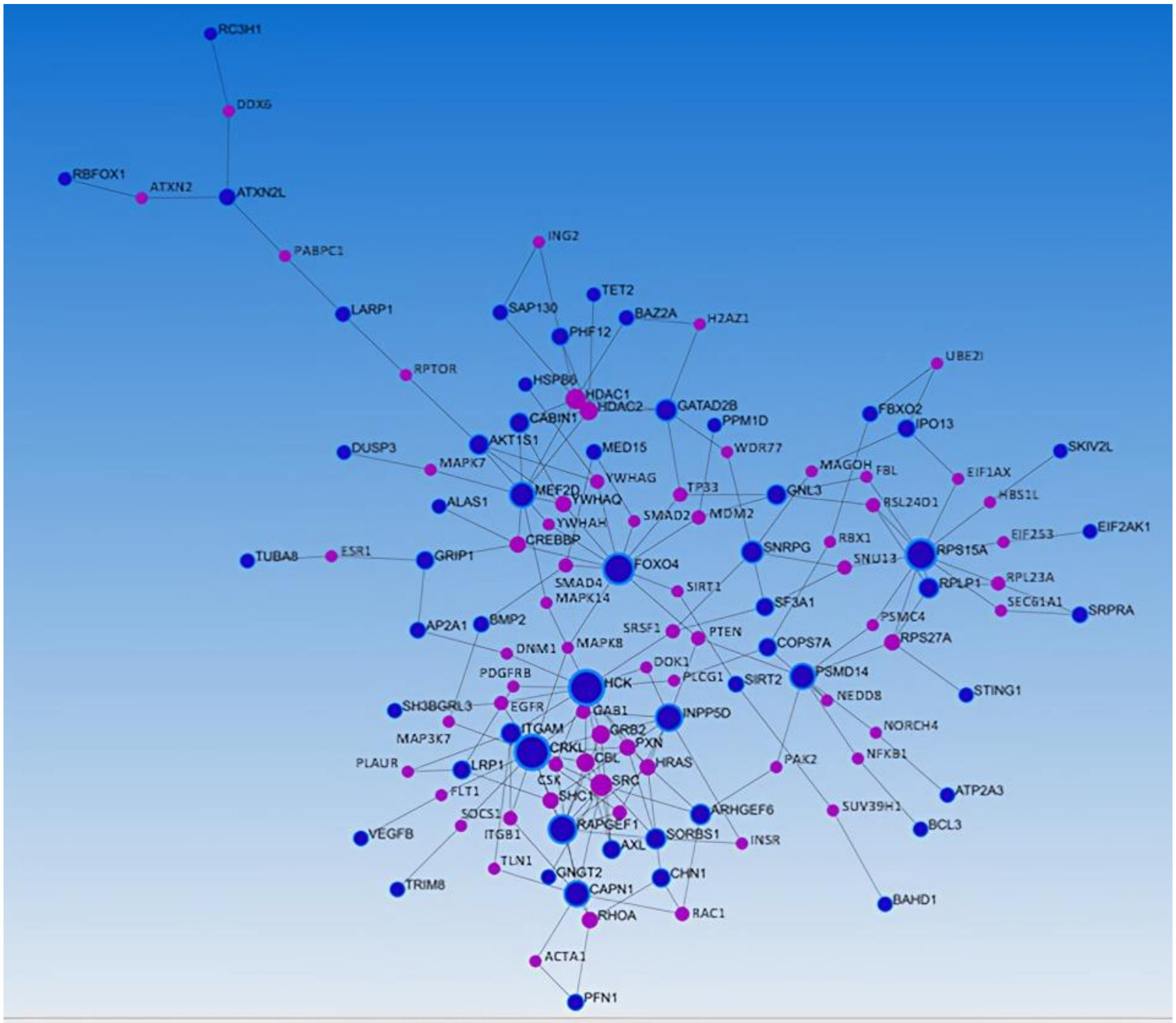

Supplementary Figure S2. Raw image protein-protein interactions (PPI) among DEGs, a PPI network was generated with the Search Tool for Retrieval of Interacting Genes/Proteins (STRING) interactome database, using the NetworkAnalyst 3.0 visual analytic platform (https://www.networkanalyst.ca/) before network image being created using BioRender to enhance visual quality.
